# Healthcare-Associated Infections-Related Bacteriome and Antimicrobial Resistance Profiling: Assessing Contamination Hotspots in a Developing Country Public Hospital

**DOI:** 10.3389/fmicb.2021.711471

**Published:** 2021-08-16

**Authors:** Aline Fernanda Rodrigues Sereia, Ana Paula Christoff, Giuliano Netto Flores Cruz, Patrícia Amorim da Cunha, Guilherme Cezar Kniphoff da Cruz, Daniela Cristina Tartari, Caetana Paes Zamparette, Taise Costa Ribeiro Klein, Ivete Ioshiko Masukawa, Clarice Iomara Silva, Maria Luiza Vieira e Vieira, Mara Cristina Scheffer, Luiz Felipe Valter de Oliveira, Thaís Cristine Marques Sincero, Edmundo Carlos Grisard

**Affiliations:** ^1^Department of Microbiology, Immunology and Parasitology, Federal University of Santa Catarina, Florianópolis, Brazil; ^2^BiomeHub, Florianópolis, Brazil; ^3^Department of Clinical Analysis, Federal University of Santa Catarina, Florianopólis, Brazil; ^4^Polydoro Ernani de São Thiago University Hospital, Federal University of Santa Catarina, Florianópolis, Brazil

**Keywords:** healthcare-associated infections, antimicrobial resistance, bacteriome, microbiome, 16S rRNA gene sequencing, beta-lactamases, healthcare workers, hospital environment

## Abstract

Hospital-built environment colonization by healthcare-associated infections-related bacteria (HAIrB) and the interaction with their occupants have been studied to support more effective tools for HAI control. To investigate HAIrB dynamics and antimicrobial resistance (AMR) profile we carried out a 6-month surveillance program in a developing country public hospital, targeting patients, hospital environment, and healthcare workers, using culture-dependent and culture-independent 16S rRNA gene sequencing methods. The bacterial abundance in both approaches shows that the HAIrB group has important representativeness, with the taxa Enterobacteriaceae, *Pseudomonas*, *Staphylococcus*, *E. coli*, and *A. baumannii* widely dispersed and abundant over the time at the five different hospital units included in the survey. We observed a high abundance of HAIrB in the patient rectum, hands, and nasal sites. In the healthcare workers, the HAIrB distribution was similar for the hands, protective clothing, and mobile phones. In the hospital environment, the healthcare workers resting areas, bathrooms, and bed equipment presented a wide distribution of HAIrB and AMR, being classified as contamination hotspots. AMR is highest in patients, followed by the environment and healthcare workers. The most frequently detected beta-lactamases genes were, *bla*_SHV–like_, *bla*_OXA–__23__–like_, *bla*_OXA–__51__–like_, *bla*_KPC–like_, *bla*_CTX–M–__1_, *bla*_CTX–M–__8_, and *bla*_CTX–M–__9_ groups. Our results demonstrate that there is a wide spread of antimicrobial resistance due to HAIrB in the hospital environment, circulating among patients and healthcare workers. The contamination hotspots identified proved to be constant over time. In the fight for patient safety, these findings can reorient practices and help to set up new guidelines for HAI control.

## Introduction

Healthcare-associated infections (HAI) are important public health threats of particular concern in the developing world, requiring continuous monitoring and the improvement of surveillance programs (Allegranzi et al., 2011; WHO, 2011).

HAI can seriously affect patient’s health, promoting long-term hospital stays and increasing mortality, besides rising costs for the healthcare system (Osme et al., 2020). Also, the combination of fast human mobility around the world with selective pressure by overuse and misuse of antibiotics in human and food-producing animals along with the difficulties in adopting simple control measures, form the perfect system to ensure the spread of Multidrug-Resistant (MDR) bacteria (Hawkey and Jones, 2009; Medina and Pieper, 2016).

There are many shreds of evidences that the hospital environment and healthcare workers (HCW) are key players on the large-scale dissemination of HAI-related MDR bacteria (Otter et al., 2013; Facciolà et al., 2019; Shams et al., 2019). Microbiome studies using high-throughput sequencing (HTS) methodologies have been conducted in health institutions (Lax et al., 2017; Christoff et al., 2020) and can elucidate important questions about HAI. These studies have been especially interested in the profile and interaction of microbial communities of the built environment, in addition to understanding the role of healthcare setting actors (patients, HCW, and the environment) in HAI spread. In this scenario, adoption of surveillance programs based on new technologies associated with the rational use of antimicrobials can allow effective HAI control management (Fournier et al., 2013; Tang et al., 2017; Christoff et al., 2019).

Shortly, these consolidated data could promote new tools for monitoring and adapting health institution’s indoor environments and guidance to health teams’ behavior, ensuring the patient safety.

To better understand the antimicrobial resistance (AMR) profile and the dynamics of HAI-related bacteria (HAIrB) communities in the healthcare setting we carried out the *“Healthcare-associated Infections Microbiome Project*” (HAIMP), a 6-month surveillance program in a typical developing country public hospital, targeting patients, the hospital environment and the healthcare workers, using culture-dependent (CD) and culture-independent (CI)16S rRNA gene sequencing methods.

## Materials and Methods

### Study Design and Sample Collection

The HAIMP was carried out at a tertiary teaching hospital (Professor Polydoro Ernani de São Thiago University Hospital—HU, Federal University of Santa Catarina—UFSC, Florianópolis/SC, Brazil) and was approved by the UFSC Human Research Ethics Committee (Number 32930514.0.0000.0121).

Between April and September of 2015, 180 sampling sites distributed in six hospital units: emergency ward (EMG), internal medicine ward (IMW), surgical ward (SUW), general surgery unit (GSU) and intensive care unit A and B (ICU-A and ICU-B), were sampled monthly from patients (PT: rectal, nasal and hands swabs; *n* = 198), hospital environment (HE: high-touch surfaces; *n* = 666) and healthcare workers (HCW: hands, mobile phone and protective clothing—white coat or scrubs; *n* = 216) (Table 1 and Supplementary Table 1). A total of 1,080 samples were collected in duplicate for CD and CI analysis, totaling 2.160 samples (Supplementary Figure 1).

**TABLE 1:** Sources and sampling points assessed using culture-dependent (CD) and culture-independent (CI) methods.

**Source**	**Sampling points**	**Hospital units**						**Total**
		**SUW**	**GSU**	**IMW**	**EMG**	**ICU-A**	**ICU-B**	**(%)**
Patient	Rectum, nasal cavity and hands	0	36	36	54	36	36	198 (18.3%)
Healthcare workers	Hands, mobile phone and protective clothing	36	36	36	36	36	36	216 (20.0%)
High-touch surfaces of hospital environment	Bed equipment, bed bathroom, unit bathroom, healthcare workers workstation and resting room, medical equipment in common use, hand hygiene devices, surgical equipment	108	84	90	144	114	126	666 (61.7%)
	Total *N* (%)	144 (13.3%)	156 (14.4%)	162 (15.0%)	234 (21.7%)	186 (17.2%)	198 (18.4%)	1.080 (100.0%)

*EMG, emergency ward; IMW, internal medicine ward; SUW, surgical ward; GSU, general surgery unit; ICU-A and ICU-B, intensive care unit A and B.*

All participants were initially informed about the study aims and procedures. Sampling was carried out upon a signature of an informed consent.

For patients, inclusion criteria were (1) long-term hospitalized patients with or without report of HAI-related infection and, (2) agreement to sign the informed consent. Patients who agreed to participate of the study answered a specific questionnaire used for data collection; medical records were also consulted in order to obtain clinical data. For HCW, inclusion criteria were (1) must be staff/employee of the university hospital and (2) agreement to sign the informed consent. Undergraduate and graduate students were not included among HCW. Environmental samples were obtained from the same high-touch surfaces throughout the study (each month the beds collected changed according to the hospitalized patients who agreed to participate of the study, however, keeping the sampling site types).

For CD analysis, sampling was performed using Amies agar gel-containing swabs (Copan Inc., Italy) and stored at 4°C until processing, while for CI analysis, sampling was performed using a sterile cotton swab and stored at room temperature until processing. The samples for CI and CD analyses were simultaneously collected (side-by-side swabs), transported to the laboratory and processed within 24 h.

### Culture-Independent Analysis

Bacterial DNA from CI samples was obtained using a thermal and cell lysis protocol followed by purification with a magnetic beads protocol (AMPure XP Magnetic Beads, Beckman Coulter, United States). A negative extraction control was included in each DNA extraction batch.

### Culture-Dependent Analysis

Culture-dependent analysis exclusively focused on Gram-negative bacteria (GNB). Amies agar gel-containing collected swabs were inoculated in Brain Heart Infusion (BHI) broth (BD, United States), incubated for 12–18 h at 36°C (± 1°C) before plating on selective MacConkey agar (BD, United States), following incubation (18–24 h, 36°C ± 1°C). Different morphotypes of Colony-Forming Units (CFU) were isolated and transferred onto new MacConkey agar plates. Identification and antimicrobial susceptibility test (AST) of each isolate were performed using Vitek^®^ 2 (BioMérieux Inc., United States) GN ID and AST-N239 cards according to the manufacturer’s instructions. Based on the AST results, the isolates were then classified as “not multidrug-resistant” (Not MDR) or “multidrug-resistant” (MDR), according to the acquired resistance classification (Magiorakos et al., 2011). DNA from all GNB isolates was extracted as described above and quantified using Qubit dsDNA BR Assay Kit (Invitrogen, United States).

### High-Throughput Sequencing of V3/V4 16S rRNA

Bacterial identification of CI and CD analyzes was performed by high-throughput sequencing of V3/V4 16S rRNA using primers 341F and 806R (Wang and Qian, 2009; Caporaso et al., 2012). Amplicon sequencing library preparation and sequencing was carried out as previously described (Christoff et al., 2020; Cruz et al., 2021), in a two-step equivolumetric PCR protocol: first PCR uses V3/V4 universal primers containing a partial Illumina adaptor, based on TruSeq structure adapter (Illumina Inc., CA, United States) that allows a second PCR with the indexing sequences. All PCR reactions were performed in triplicates and with a negative reaction control. The second PCR reactions were cleaned up using AMPureXP beads (Beckman Coulter, IN, United States) and an equivalent volume of each sample was added in the sequencing library pool. The final DNA concentration of the library pool was estimated with Quant-iT Picogreen dsDNA assays (Invitrogen, MA, United States), and then diluted for accurate qPCR quantification using KAPA Library Quantification Kit for Illumina platforms (KAPA Biosystems, MA, United States). The 16S rRNA libraries sequencing pool was adjusted to a final concentration of 11 pM and sequenced using the MiSeq Sequencing System (Illumina Inc., CA, United States) with the V2 kit, 300 Cycles, single-end sequencing.

The sequenced reads obtained were subjected to demultiplexing processes along with primers and adapters trimming. Application of quality filters was performed using a bioinformatics pipeline previously described (Cruz et al., 2021). Reads were analyzed with the Deblur package v.1.1.0 (Amir et al., 2017) to remove possible erroneous reads and identical sequences were grouped into oligotypes (clusters with 100% identity). The sequence clustering with 100% identity provides a higher resolution for the amplicon sequencing variants (ASVs), also called sub-OTUs (sOTUs) (Knight et al., 2018)—herein denoted as oligotypes. Then, VSEARCH 2.13.6 (Rognes et al., 2016) was used to remove chimeric amplicons. Additional filters were used to remove oligotypes below the frequency cutoff of 0.2% in the final number of counts/sample. A negative control filter, consisting in removal of oligotypes recovered in DNA extraction and PCR reactions negative controls was also employed, considering that the number of “contaminant” reads are no greater than two times their respective counts in the samples. The remaining oligotypes in the samples were used for taxonomic assignment with the BLAST tool (Altschul et al., 1990) against a reference in-house genome database (encoderef16s_rev6_190325, BiomeHub, SC, Brazil). This database contains 11,750 sequences from complete and draft bacterial genomes of medical relevance retrieved from NCBI, representing 1,843 different bacterial taxonomies.

Taxonomies are assigned to each oligotype using a lowest common ancestor (LCA) algorithm. If more than one reference can be assigned to the same oligotype with equivalent similarity, the taxonomic assignment algorithm leads the taxonomy to the lowest level of possible unambiguous resolution (genus, family, order, class, phylum, or kingdom), according to similarity thresholds previously established (Yarza et al., 2014).

The resulting oligotype tables were processed and normalized as previously described (Cruz et al., 2021). Oligotype sequences served as input for FastTree 2.1 software (Price et al., 2010) to construct phylogenetic trees and allow beta-diversity analysis with UniFrac distances (Lozupone and Knight, 2005). Additional analyses were performed using R (version 3.6.0) and the Phyloseq package (McMurdie and Holmes, 2013). Alpha diversity analysis was performed using the Phyloseq “plot_richness” function.

For some analyzes we focus on a bacteria group that have been largely related to HAI in developing countries, that we call HAIrB: *Acinetobacter baumannii*, *Burkholderia cepacia*, *Escherichia coli*, *Enterococcus faecium*, *Enterococcus faecalis*, *Enterobacter*, Enterobacteriaceae, *Staphylococcus aureus*, *Klebsiella pneumoniae*, *Pseudomonas aeruginosa*, *Proteus mirabilis*, and *Serratia marcescens*.

### Screening of Genes Encoding for Beta-Lactamases by qPCR

For CI and CD analyzes, a panel of the most important beta-lactamases (BL) genes in the Brazilian scenario (*bla*_OXA–__23__–like_, *bla*_OXA–__48__–like_, *bla*_OXA–__51__–like_, *bla*_OXA–__58__–like_, *bla*_OXA–__72__–like_, *bla*_OXA–__143__–like_, *bla*_CTX–M–__1_ group, *bla*_CTX–M–__2_ group, *bla*_CTX–M–__8_ group, *bla*_CTX–M–__9_ group, *bla*_CTX–M–__25_ group, *bla*_KPC–like_, *bla*_SHV–like_, *bla*_NDM–like_, *bla*_IMP–like_, *bla*_VIM–like_, *bla*_GES–like_, and *bla*_SPM–like_) was carried out by qPCR using specific primers and hydrolysis probes in a duplex or triplex configuration. Reactions were performed in a 10 μL final volume, containing 2 μL of the same previously sequenced DNA samples, 0.2 U Platinum Taq, 1 × Buffer, 3 mM MgCl_2_, 0.4 mM dNTP, 0.016 mM ROX^*TM*^ (Invitrogen, MA, United States), 0.2 μM of each forward and reverse specific primer, 0.15 μM of Cy5^®^, 0.1 μM of HEX^*TM*^ and/or 0.05 μM of FAM^*TM*^ labeled probes (Applied Biosystems, MA, United States). The assays were carried out in technical triplicates containing a negative reaction control and positive controls for each resistance gene (characterized strains containing the gene of interest). The qPCR amplifications were performed on an ABI 7500 Fast Real-Time PCR System (Applied Biosystems, CA, United States), using the following thermal conditions: 95°C for 10 min, 35 cycles of 95°C for 15 s, 60°C for 30 s and 72°C for 30 s.

## Results

### Contamination Hotspots in the Healthcare Setting

Aiming to determine the bacterial contamination hotspots within the hospital, the total read count (library size) for bacteria corresponding to the HAIrB group (CI approach), as well as GNB isolation (CD approach) were comparatively evaluated.

**FIGURE 1:**
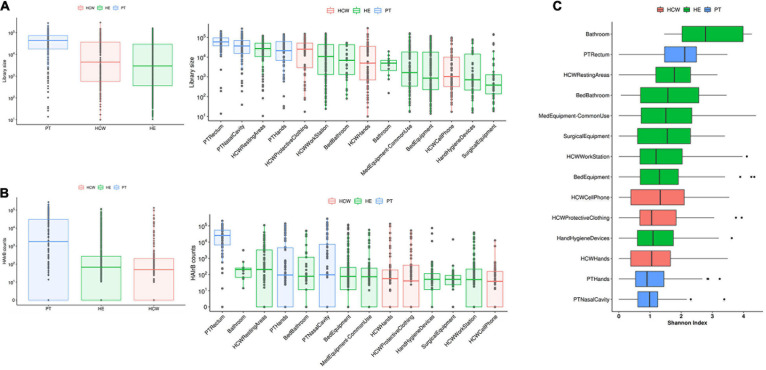
Culture-independent analyses of HAIB in a tertiary public hospital. **(A)** Total bacterial sequenced reads (library size) in *log*_10_ scale represented by boxplots with the median bacterial distribution for patients (PT), healthcare workers (HCW) and healthcare environment (HE)-left panel—and corresponding specific sites—right panel. **(B)** Total sequenced reads corresponding to HAI-related bacteria (HAIrB counts) for PT, HCW and HE—left panel—and corresponding specific sites—right panel. **(C)** Shannon alpha-diversity index for PT, HCW and HE specific sites.

**FIGURE 2:**
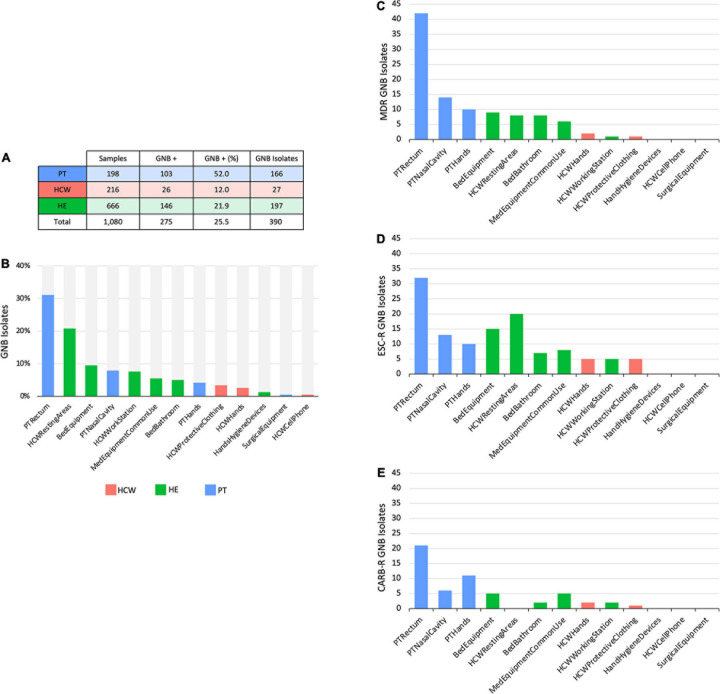
Culture-dependent analyses of HAIrB in a tertiary public hospital. **(A)** Number of collected samples from patients (PT), healthcare workers (HCW) and healthcare environment (HE), samples positive for at last one Gram-negative bacteria (GNB) and GNB Isolates recovered from them. **(B)** GNB isolates recovered from PT, HCW and HE specific sites. **(C)** Number of multidrug-resistant (MDR), **(D)** extended-spectrum cephalosporins resistant (ESC-R) and **(E)** carbapenem resistant (CARB-R) GNB from PT, HCW, and HE specific sites.

Patients (PT) have the highest median read count, HAIrB abundance and isolation rate of GNB, mostly related to the PT rectum samples if compared to hands and nasal samples, as expected (Figures 1, 2).

In the HCW, despite the high abundance of total bacteria in the protective clothing, the median distribution of HAIrB was similar for the hands, clothing and mobile phones.

Concerning the hospital environment, the HCW resting areas stand out as the most contaminated, with a high count of both total bacteria and HAIrB. It is also noticeable that bathrooms of the units, regardless of whether they are for collective use or restricted to beds, as well as the bed equipment and medical equipment in common use by the hospital units, presented a wide distribution of HAIrB. It is noteworthy to mention that the units’ bathrooms had the highest values of the Shannon diversity index of HAIrB, followed by PT rectum and HCW resting areas (Figure 1C). Areas or equipments considered to be cleaner such as the HCW workstations used during medical and nursing procedures, or surgical equipment restricted to GSU, revealed substantial contamination by HAIrB despite having lower amounts of total bacteria.

The CD approach showed a similar distribution pattern of GNB isolates, indicating a higher number of isolates obtained from PT, HCW resting areas, and bed equipment compared to surgical equipment and HCW mobile phones, which showed a reduced number of GNB isolates (Figures 2A,B). The AMR profiles corroborate these contamination hotspots, highlighting PT and their surroundings, in addition to the HCW resting areas, HCW workstation, and medical equipment of common use (Figures 2C–E). All these areas presented MDR, as well as extended-spectrum cephalosporins resistant (ESC-R), and carbapenem resistant (CARB-R) GNB.

**FIGURE 3:**
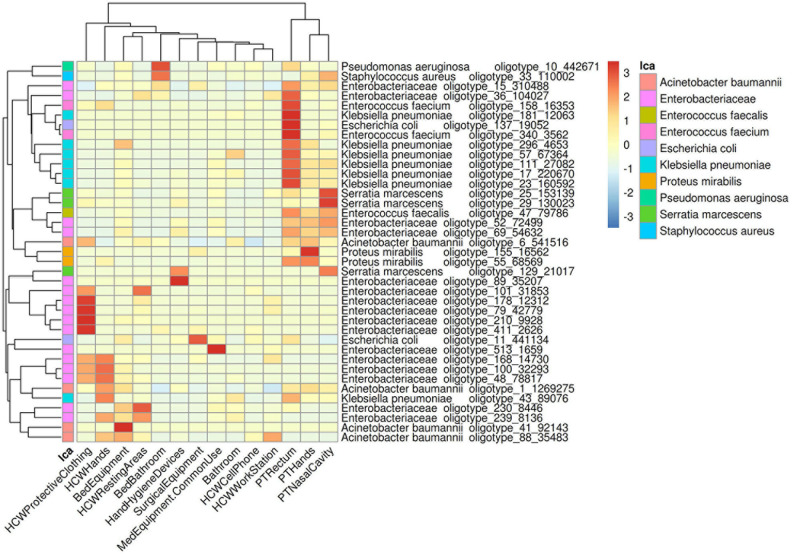
Dispersion of HAIrB in the healthcare setting. Heatmap of HAIrB oligotypes average abundance in specific sites from patients (PT), healthcare workers (HCW) and healthcare environment (HE). Oligotypes are identified with a unique crescent code and its count number (oligotype_code_count). Bacterial taxonomies were added near the oligotype name to facilitate visualization. Red color represents highest abundance for HAIrB oligotypes in the specific sites.

In order to understand the dispersion of HAIrB within the healthcare setting, the average abundance of the oligotypes corresponding to HAIrB obtained using the CI approach were used to plot a heat map (Figure 3). There is clear and distinct grouping of PT samples, demonstrating a higher frequency of oligotypes shared between the different sampling sites (nasal cavity, rectum, and hands) like olygotype_47 from *E. faecalis* and oligotype_69 from Enterobacteriaceae. A second grouping, containing samples of HCW protective clothing and hands, HCW resting areas, and bed equipment, was also identified. The oligotype_239 identified as Enterobacteriaceae was tracked in bed equipment, HCW hands and HCW resting areas. Oligotypes_169, 100 and 48 corresponding to Enterobacteriaceae were found in the HCW hands and protective clothing, while oligotype_101 was found in the HCW hands and resting areas. It was also possible to find more widely dispersed oligotypes, such as the oligotype_88, identified as *A. baumannii*, abundantly dispersed in bed equipment, HCW hands and HCW workstations. Oligotype_43, identified as *K. pneumoniae*, was screened in HCW hands and in PT rectum. The oligotype_6 identified as *A. baumannii* was found in PT hands and rectum and with similar abundance in HCW protective clothing. HAIrB dispersion was also evaluated comparatively between CI and CD approaches (Supplementary Figure 2), showing detection of several overlapping oligotypes in many CI and CD correspondent samples.

For educational purposes and to promote that HAI prevention control measures be discussed, the data of contamination hotspots was shown to the HCW teams of each hospital unit and to the infection control committee using heatmaps graphs created based on the hospital blueprint (Supplementary Figures 3–7). The reads count of each sampling points collected (environment, HCW and PT) were plotted on the hospital blueprint, showing a larger and redder heat point according to the amount of HAI related bacteria (expanded HAIrB group, Supplementary Table 2). The recurrent contamination hotspots over the 6-month surveillance program in certain areas can be easily visualized and mapped by the staff using this graph model.

### Bacteriome Profile Along Time and Location

**FIGURE 4:**
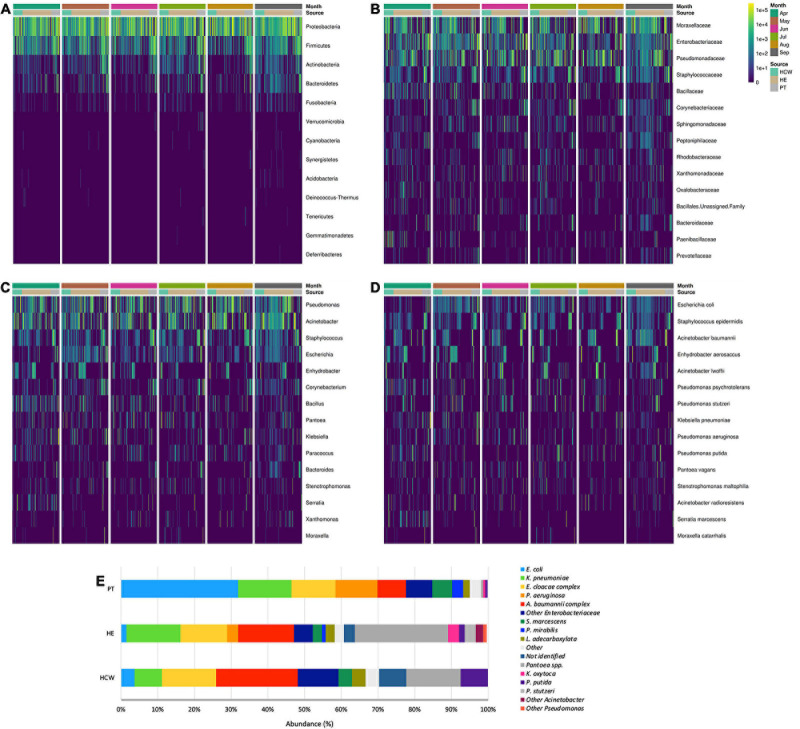
General bacterial composition and abundance recovered along the six-month surveillance program. Bacterial identification obtained for culture independent approach showing **(A)** phylum, **(B)** family, **(C)** genus and **(D)** species taxa along time (months), separated by patients (PT), healthcare workers (HCW) and healthcare environment (HE) sites. **(E)** bacterial identification obtained for culture-dependent approach separated by PT, HCW and HE sites.

The abundance of bacteria in CI and CD approaches showed that the HAIrB group has an important representativeness (Figure 4), with the taxa Enterobacteriaceae, *Pseudomonas*, *E. coli*, *S. epidermidis*, and *A. baumannii* widely dispersed and abundant over the 6-month surveillance program. Supplementary Figure 8 shows an agreement analysis to verify the different GNB identification techniques used (Vitek^®^ 2 and HTS of 16S rRNA V3/V4 gene) in CD approach. The results demonstrate an excellent agreement between the different methods.

**FIGURE 5:**
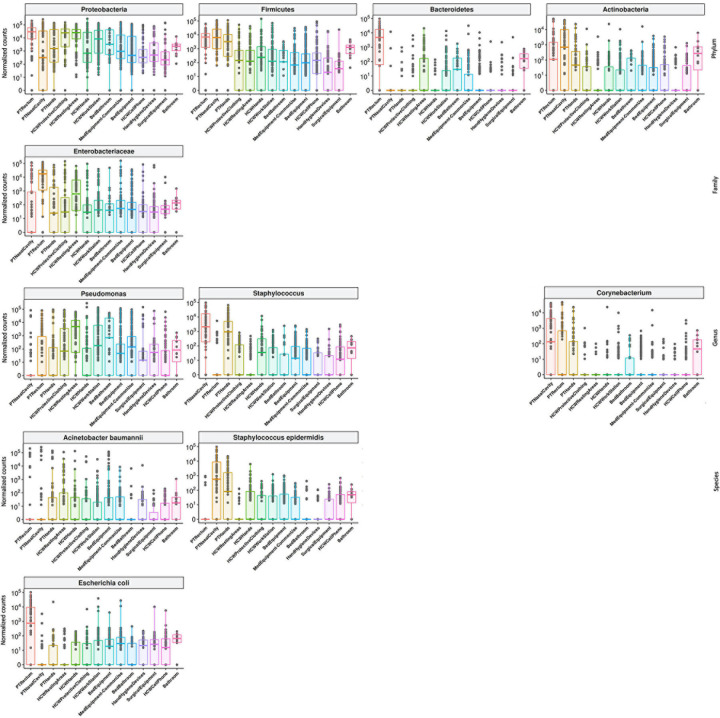
Most abundant and dispersed taxons in the healthcare setting. Bacterial sequenced reads (normalized counts) in *log*_10_ scale for the most abundant and widely dispersed taxa represented by boxplots with the median bacterial distribution for specific sites from patients (PT), healthcare workers (HCW) and healthcare environment (HE).

High-throughput sequencing data (CI approach) showed that Proteobacteria phylum was the most abundant, followed by Firmicutes, Actinobacteria, and Bacteroidetes (Figure 4). Proteobacteria and Firmicutes, notably the phyla with most HAIrB members, is widely dispersed over time in PT, HCW and HE. The phylum Proteobacteria was mostly represented by Enterobacteriaceae, *Pseudomonas*, *A. baumannii*, and *E. coli*, while *Staphylococcus*, in particular *S. epidermidis*, was the most representative of the phylum Firmicutes. Bacteroidetes were identified mainly in PT rectum and bathrooms, while Actinobacteria, although also dispersed, was identified with greater abundance and frequency in PT samples (nasal cavity, rectum and hands) and bathrooms, with *Corynebacterium* as the most important taxon. Some HAIrB were found in greater abundance in specific sites, such as *K. pneumoniae* and *P. aeruginosa*, widely dispersed over time in PT rectum and bathrooms (Figure 5).

**FIGURE 6:**
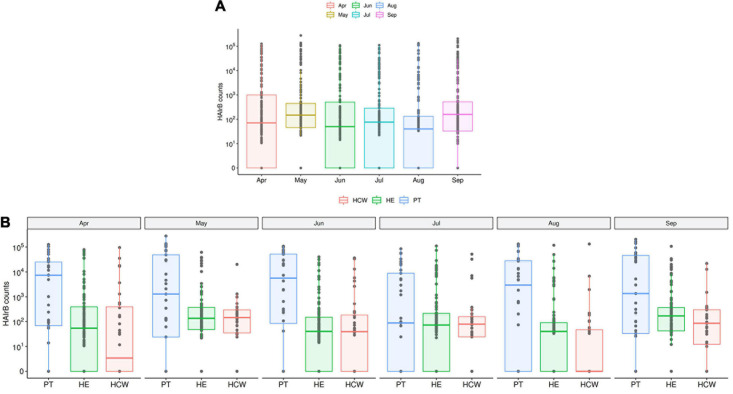
HAI related bacteria recovered along the six-month surveillance program. **(A)** Total sequenced reads in *log*_10_ scale corresponding to HAI-related bacteria (HAIrB counts) represented by boxplots with the median bacterial distribution along six-month study and **(B)** separated by patients (PT), healthcare workers (HCW) and healthcare environment (HE) sites.

**FIGURE 7:**
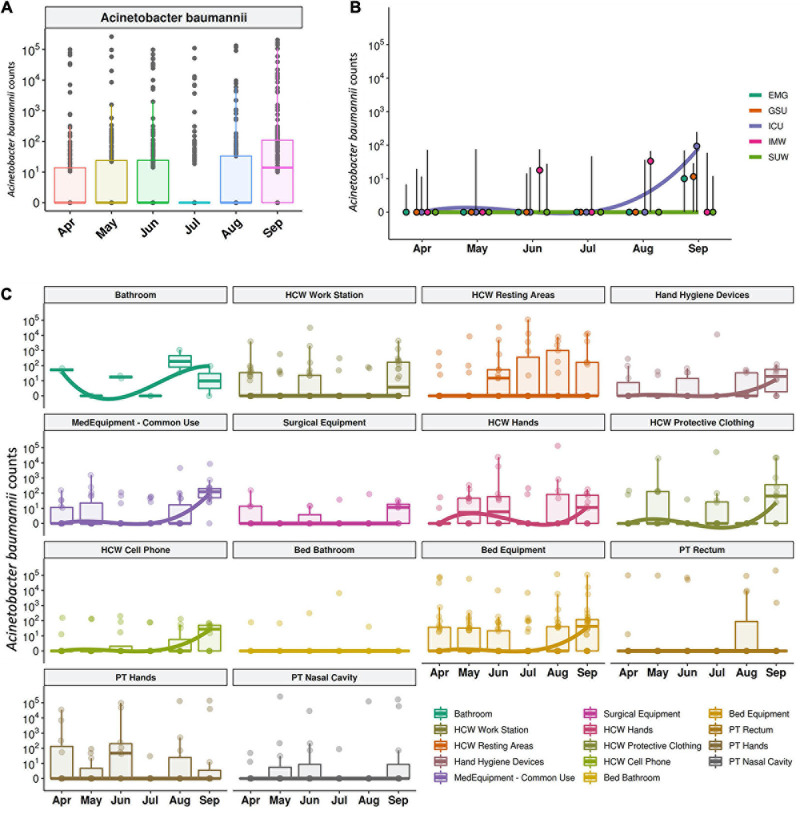
*Acinetobacter baumanni* along the time and healthcare setting sites. **(A)** Total sequenced reads in *log*_10_ scale corresponding to *A*. *baumanni* represented by boxplots with the median bacterial distribution along six-month study. Longitudinal profiles of *A*. *baumannii* **(B)** along time in the different hospital units and **(C)** for patients (PT), healthcare workers (HCW) and healthcare environment (HE) specific sites. Boxplots are ordered by median values, marked with a tendency line along the months.

The median abundance of HAIrB for CI approach was higher in May and September and this increase is more related to bacterial load in HCW and healthcare environment (HE) (Figure 6). In fact, in September the hospital experienced an outbreak by *A. baumannii*, that could be seen in the CI data, with increased abundance of this HAIrB on this month (Figure 7A), as well as in CD data (isolation of *A. baumannii* increased 42,5%). In May the *E. coli* abundance increased, but without official reports of an outbreak. Longitudinal profile shows that there was an increase of *A. baumannii* in ICU along August and September (Figure 7B). This increase pattern can be seen in some specific locations, such as bed equipment, medical equipment in common use and HCW samples (Figure 7C). By tracking the oligotypes corresponding to *A. baumannii* and their distribution in the hospital units over time, a widely dispersed oligotype was found: oligotype_1. It is noteworthy that oligotype_1 was the most abundantly found considering all the analyzed samples. An increased abundance of this oligotype was found in ICU, EMG and IMW in September, in agreement with the outbreak reported.

**FIGURE 8:**
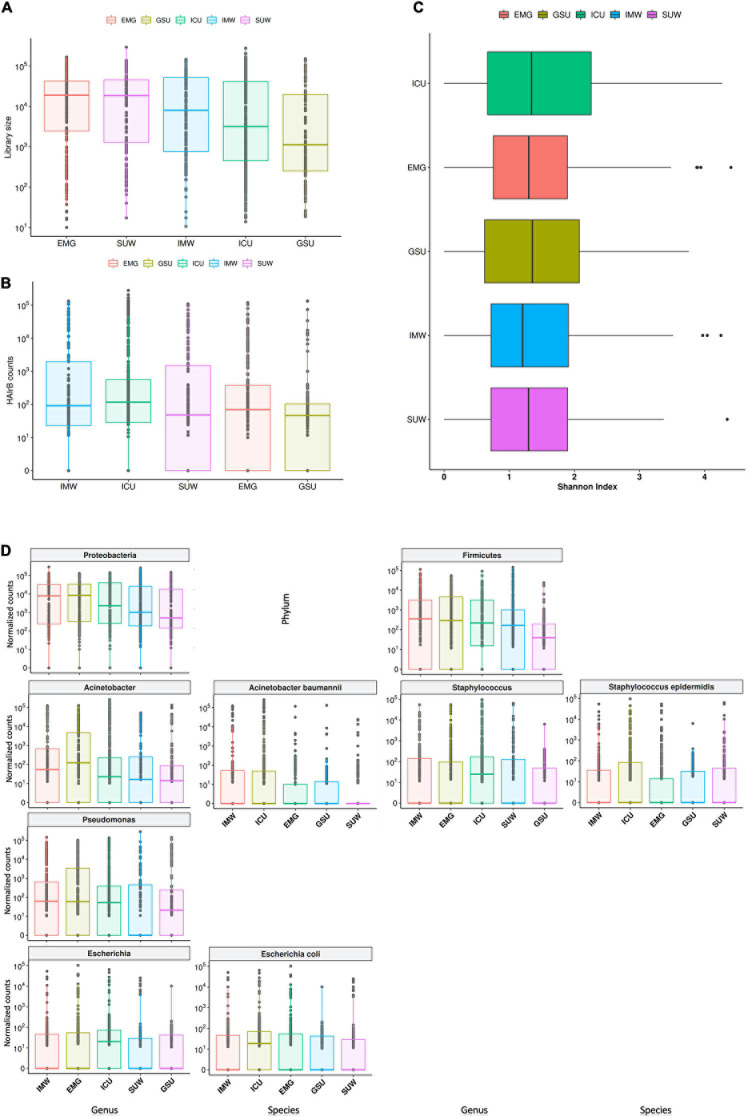
Hospital unit’s profile. **(A)** Total bacterial sequenced reads (library size) in *log*_10_ scale represented by boxplots with the median bacterial distribution for intensive care unit (ICU), internal medicine ward (IMW), surgical ward (SUW), emergency ward (EMG), and general surgery unit (GSU). **(B)** Total sequenced reads corresponding to HAI-related bacteria (HAIrB counts) for the five hospital units. **(C)** Shannon alpha diversity index for the five hospital units. **(D)** Bacterial sequenced reads (normalized counts) in *log*_10_ scale for the most abundant and widely dispersed taxa represented by boxplots with the median bacterial distribution for the five hospital units.

The hospital units had high loads of total bacteria, with EMG showing the highest median value and GSU with the lowest median (Figure 8A). However, it is possible to observe that the ICU, despite the lower median of total bacteria than other units, showed the highest HAIrB contamination in most samples analyzed, along with IMW, GSU and SUW have smaller and similar medians of HAIrB (Figure 8B).

The different hospital units did not present different Shannon diversity index values (Figure 8C). All hospital units had a higher abundance of Proteobacteria and Firmicutes (Figure 8D). *Acinetobacter*, *Pseudomonas*, *Escherichia* and *Staphylococcus* were the most abundant and dispersed genera throughout the hospital units.

**FIGURE 9:**
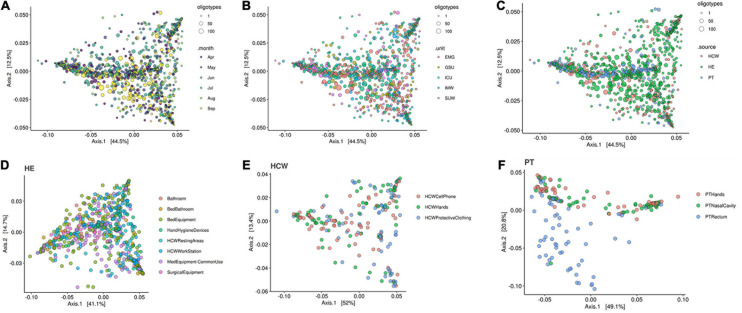
Beta-diversity analysis for the healthcare setting. Beta-diversity weighted UniFrac analysis represented by PCoA plots clustered by **(A)** time in months, **(B)** the five hospital units, **(C)** patients (PT), healthcare workers (HCW) and healthcare environment (HE) sites, **(D)** HE specific sites, **(E)** HCW specific sites and **(F)** PT specific sites. Patient samples, especially PT rectum, were the only that showed closer clusters—middle blue dots in panel (C) and bottom blue dots in panel (F). Circle sizes represent the oligotypes counts. ICU, intensive care unit; IMW, internal medicine ward; SUW, surgical ward; EMG, emergency ward; GSU, general surgery unit.

The beta-diversity weighted UniFrac analysis (Figure 9) did not show cluster profiles related to time, hospital units, HCW or HE sites. As expected, patient rectal samples showed a differential dispersal pattern when compared to other sampling sites (Figures 9C,F).

### Antimicrobial Resistance Profile

**FIGURE 10:**
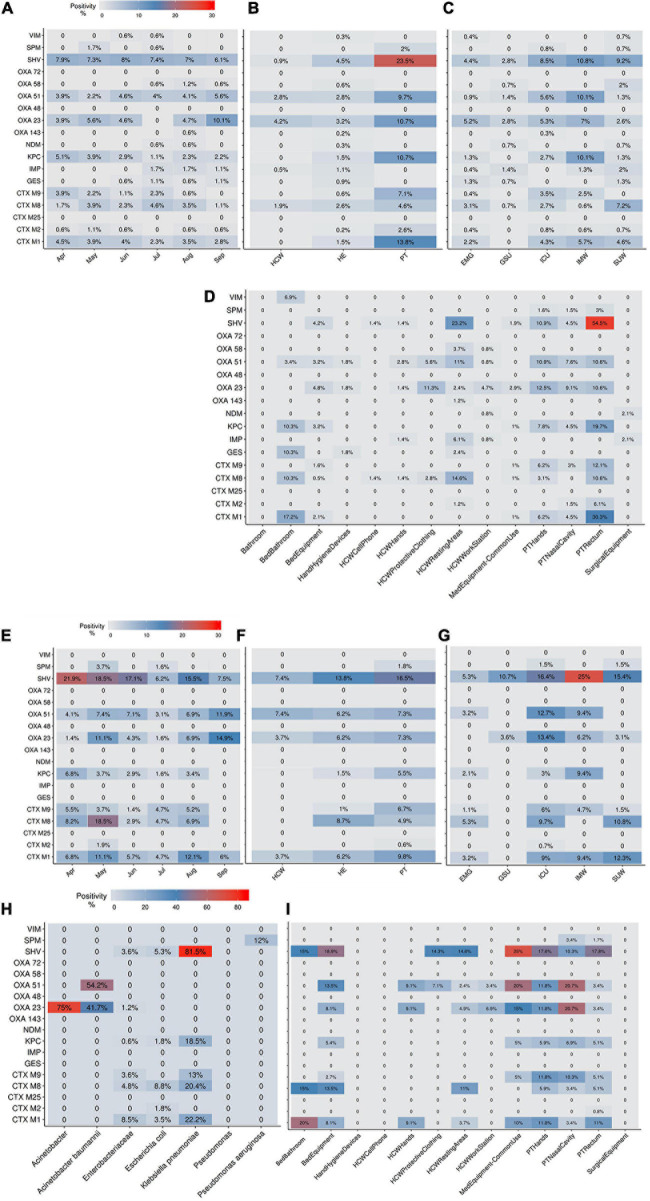
Beta-lactamases genes frequencies for culture-independent and culture-dependent approaches. **(A–D)** Beta-lactamases (BL) genes frequencies for culture-independent approach **(A)** along six-months study, **(B)** in patients (PT), healthcare workers (HCW) and healthcare environment (HE), **(C)** in five hospital units and **(D)** in PT, HCW and HE specific sites. **(E–I)** BL genes frequencies for culture-dependent approach **(E)** along six-months study, **(F)** in PT, HCW and HE, **(G)** in five hospital units, **(H)** in specific bacteria taxa and **(I)** in PT, HCW and HE specific sites. Higher BL genes frequencies are shown in red. ICU, intensive care unit; IMW, internal medicine ward; SUW, surgical ward; EMG, emergency ward; GSU, general surgery unit.

**FIGURE 11:**
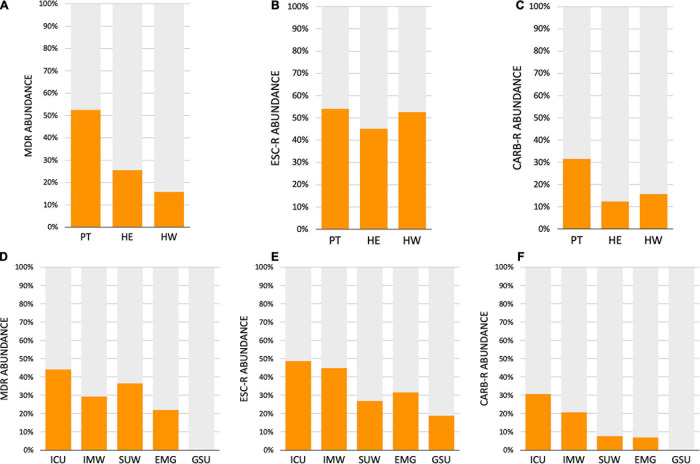
Acquired resistance classification for Gram-negative bacteria. **(A–C)** Abundance of Gram-negative bacteria (GNB) isolated from patients (PT) healthcare workers (HCW) and healthcare environment (HE), classified as **(A)** multidrug-resistant (MDR), **(B)** extended-spectrum cephalosporins resistant (ESC-R) and **(C)** carbapenem resistant (CARB-R). **(D–F)** Abundance of GNB isolated from five hospital units, classified as **(A)** MDR, **(B)** ESC-R and **(C)** CARB-R. ICU, intensive care unit; IMW, internal medicine ward; SUW, surgical ward; EMG, emergency ward; GSU, general surgery unit.

The AMR profile investigation focused on the identification of beta-lactamases (BL) using AST for GNB isolates (CD approach) and BL genes for CI and CD-derived samples. Figure 10 show the frequencies of BL coding genes detected by both approaches over time, hospital units, specific sites, and specific HAIrB. Figures 2, 11 shows the GNB isolates abundance and their respective MDR, ESC-R and CARB-R profiles.

Our results indicate that AMR is higher among PT than HE and HCW samples. Corroborating the findings for contamination hotspots, the AMR was higher in HCW resting areas, bed equipment, bed bathroom and medical equipment in common use. Interestingly, protective clothing presented higher AMR than the hands among HCW, while in PT, AMR profiles of all sampled sites are noteworthy. In hospital units, ICU, IMW, and SUW stand out, with higher AMR. The most frequently AMR bacteria identified in the contamination hotspots were *A. baumannii*, *K. pneumoniae*, other Enterobacteriaceae (e.g., *Enterobacter cloacae*) and *E. coli*.

The most frequently found beta-lactamases genes were *bla*_SHV–like_, *bla*_OXA–__23__–like_, *bla*_OXA–__51__–like_, *bla*_KPC–like_, *bla*_CTX–M–__1_, *bla*_CTX–M–__8_, and *bla*_CTX–M–__9_. Detection of BL genes was higher among samples obtained *via* CI approach than CD, among which, some genes such as *bla*_NDM–like_, *bla*_OXA–__58__–like_, and *bla*_IMP–like_ were exclusively detected within CI samples, probably due to the increased bacterial diversity recovered in a single sample.

Supplementary Figure 9 shows the identification of BL genes in GNB isolates that presented MDR, ESC-R and CARB-R profiles, demonstrating the important contribution of this resistance mechanism in the AMR. In some antimicrobial susceptible profile GNB, BL genes were detected, demonstrating potential AMR.

### Bacteriome and HAI Risk Factors

Analysis of the medical records and the completed questionnaires of the long-term inpatients participating in this study revealed that the average age was 63.2 years (± 16.5 years), being 47.0% female and 53.0% male. The average length of hospital stay was 15.3 days, and the longer hospitalization was seen for SUW patients, with 24.3 days, followed by IMW (23.4 days), ICU-B (15.0 days), ICU-A (10.4 days) and EMG (6.8 days). Around 54.5% of these inpatients were admitted to more than one hospital unit during their stay and 65.2% undergone procedures involving at least one invasive device. Hospital records also show that 50.0% of the patients did not present HAI during the current hospitalization, 43.9% had a HAI during the current hospitalization and 6.1% were colonized by HAI-related bacteria (previous data to the samples collection of the present study).

**FIGURE 12:**
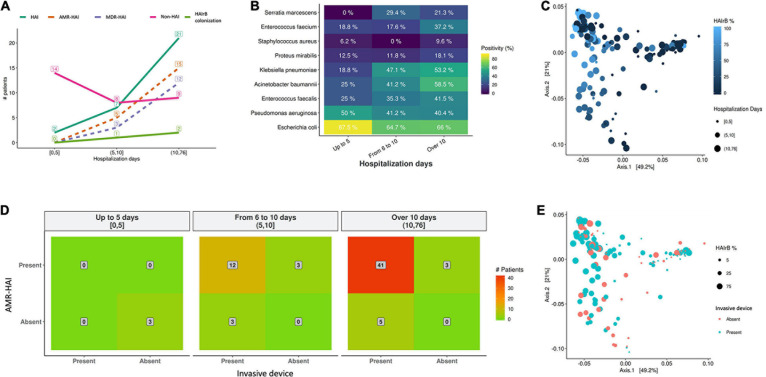
Healthcare associated infection risk factors analyses. **(A)** Number of patients diagnosed with healthcare associated infection (HAI) along the hospital stay length in days. **(B)** Positivity of healthcare associated infection related bacteria (HAIrB) identified in patients with HAI along the hospital stay length in days. **(C)** Beta-diversity weighted UniFrac analysis represented by PCoA plots clustered by the frequency of HAIrB (dark blue to light blue) identified along the hospital stay length in days (circle sizes). **(D)** Heatmap for the presence of HAI and use of at least one invasive device by patients, considering hospital stay length in days—red color represents higher number of patients (indicated in the middle of each matrix square). **(E)** Beta-diversity weighted UniFrac analysis represented by PCoA plots clustered by patients using at least one invasive device (circle sizes represent the frequency of HAIrB). HAI, healthcare associated infection; HAIrB, healthcare associated infection related bacteria; HAI-AMR, healthcare associated infection with an antimicrobial resistant bacteria involved; MDR-HAI, healthcare associated infection with a multidrug-resistant bacteria involved.

As shown on Figure 12, our data suggests that longer hospitalization stays incur in increased HAI incidence and AMR bacterial infection (Figure 12A) and increased detection of HAIrB (Figure 12B), especially *K. pneumoniae*, *A. baumannii*, *E. faecium*, *E. faecalis*, and *S. marcescens*. However, beta-diversity weighted UniFrac analysis for this correlation did not show differential clustering between hospitalization days and HAIrB proportion in patient samples (Figure 12C).

Regarding the use of invasive devices such as urinary catheters or central venous catheters, the hospital records corroborate our data and seems to indicate that longer hospital stays of these patients increases the number of patients with AMR bacterial infection (Figure 12D). However, no differential diversity profiles were observed on Beta-diversity PCoA plots for the use of invasive devices and the presence of HAIrB (Figure 12E).

## Discussion

Healthcare-associated infections, especially those involving AMR, are still a major challenge for patient safety during hospitalization. Over the years, new technologies such as high-throughput DNA sequencing have emerged, allowing deeper and precise understanding about the interaction between the hospital environmental conditions, their occupants and further associated microbiomes. The use of genomics in this sense can provide specific knowledge toward new guidelines for combating HAI, promoting and improving health. Here, a 6-month genomics surveillance program carried out in a developing country public hospital to monitor HAI-related bacteriome from patients (PT), healthcare workers (HCW) and hospital environment (HE) provide a wider and comprehensive data not obtained by traditional methods used for detection of HAIrB, offering the detection of specific MDR-related genes.

The HE bacteriome was mainly composed of Proteobacteria and Firmicutes, as formerly described (Oberauner et al., 2013; Lax et al., 2017; Chopyk et al., 2020). However, bacteriome dominant genera and species composition have less similarity than these previous hospital environment studies, who reported genera such as *Staphylococcus*, *Streptococcus*, *Corynebacterium*, *Pseudomonas*, *Propionibacterium*, *Burkholderia* and *Acinetobacter* as the most abundant and dispersed in the sample sources (Oberauner et al., 2013; Lax et al., 2017; Chopyk et al., 2020). Along geographical and environmental variability, this is also probably due to the particularities of the health institutions, especially when comparing hospitals in developed and developing countries, such as bacterial endemicity and prevalence of certain types of HAI. A study carried out in a Brazilian tertiary public hospital shows closer bacterial profiles (Christoff et al., 2020), but still with differences, mainly in the abundance of Gram-positive bacteria genera, that were less abundant in our study. The herein presented results shows the occurrence of a broad spectrum of HAIrB on the HE bacteriome, including the finding of MDR bacteria identified in patient care areas or areas used exclusively by HCW that undoubtedly increases the risk of HAI.

Considering the HAIrB and the AMR profile, some areas/equipments such as bed equipment, bed bathrooms and HCW resting areas (located within each hospital unit and used by HCW for snack and coffee or sleeping) were classified as contamination hotspots. AMR analysis supported these findings, with detection of MDR, CARB-R and ESC-R profiles and BL genes (such as *bla*_KPC–like_, *bla*_OXA–__23__–like_, *bla*_IMP–like_, *bla*_SHV–like_ *bla*_CTX–M–__1_, *bla*_CTX–M–__8_, and *bla*_CTX–M–__9_) in all of these contamination hotspots. Several studies conducted in Brazil and Latin America have confirmed the relevance of these BL genes in the AMR scenario (Jácome et al., 2016; Labarca et al., 2016; Rocha et al., 2016, 2019; da Silva et al., 2019; Romanin et al., 2019; Zagui et al., 2020). The oligotypes tracking allowed us to verify that, in general, the bacteria found in patients sites are more related to each other (different body sites) than to their surrounding environment. Similarly, the bacteria found in HCW sites, HCW resting areas and bed equipment are more related to each other, than the patients. However, several oligotypes were found to be more widely dispersed. Overlapping oligotypes were found in HCW resting areas, HCW hands, HCW protective clothing, PT bed equipment and PT rectum, suggesting a cross contamination and demonstrating possible barrier breaks and insufficient adherence to hand hygiene protocols. Another important finding tracking oligotypes was the widely dispersed *A. baumannii* oligotype_1, with an abundance increase in September in several locations and hospital units, when an outbreak was reported by the hospital. Beta-lactamases genes positivity map also demonstrates similar profiles among PT, HCW and the surfaces around them. These results are similar to those found in a recent 1 year cross-sectional study, which showed that the microbiome and AMR genes profile of ICU and NICU (neonatal ICU) maintains a close relationship with the inpatients (Christoff et al., 2020). Another study to characterize bacterial dynamics among hospital surfaces, patients and healthcare workers over the course of a year, while a new hospital became operational, demonstrated the change in bacterial diversity in the pre and post occupation periods of the hospital (Lax et al., 2017). In addition, it also showed the correlation of the patient microbiota with the environment of his room and the staff microbiota with the nurse stations (Lax et al., 2017).

New DNA sequencing-based microbiome technologies coupled to comparative sequence analysis proved to helpful in assisting health institutions to improve HAI prevention policies and practices. The risk maps based on the hospital blueprint showing HAIrB contamination hotspots, which were available to HCW teams, intended to fulfill this objective. In addition to step up HAI prevention practices that are widely disseminated among HCW, microbiome-directed discussion on the hospital layout design is necessary (Kembel et al., 2012; Stiller et al., 2016), so that patient care and daily routines do not oppose patient safety.

HAIrB contamination was high along the 6-month surveillance study in all hospital units, showing a bacterial compositional homogeneity with no beta-diversity clustering. Previous studies on microbiome of the built-environment conducted in ICU and NCIU show different results from the beta-diversity analyzes, sometimes showing compositional differences between the units, and sometimes showing that they do not differ (Ribeiro et al., 2019; Christoff et al., 2020). However, apparently when observing the data over time, with a larger sample sizes and without intentional interventional processes, point differences seem to tend to disappear, as the dynamics of the bacterial community undergoes random fluctuations over the months. Some differences in the hospital unit’s colonization profile can be related to patient’s turnover and hospital stay in each unit, as well as the severity illness of inpatients. As seen in the HAI risk factor analysis, longer hospital stay is correlated with the presence of HAI in the current hospitalization, specific HAIrB increase and invasive device use, with several large studies also documenting these correlations (Jia et al., 2019; Sahiledengle et al., 2020; Su et al., 2020). EMG with high patient’s turnover (shorter hospital stay), showed less HAIrB load compared to the total bacteria, as well as lower positivity of BL genes, MDR and CARB-R bacteria. ICU, with longer hospital stay and critical inpatients, presented high abundance of HAIrB and AMR, as well as IMW, where patients with a diagnosed HAI are preferably hospitalized or transferred. SUW, despite being a unit designed to receive patients in the pre and postoperative care, presented contamination and AMR patterns similar to the ICU and IMW, probably due to the longer hospital stay that was documented for this unit. Global low or null BL genes positivity and MDR bacteria in GSU is due to the absence of patients collected in this unit, since patients stay in the unit only for surgery and recovery.

The bacteriome of the PT rectum showed the greatest differences in alpha and beta-diversity in relation to all other body and environment sites. A healthy rectum/gut bacteriome presents high abundance of Bacteroidetes and Firmicutes and low abundance of Proteobacteria (Huttenhower et al., 2012; Lloyd-Price et al., 2016). The high abundance of Proteobacteria is related to gut dysbiosis that hospitalized patients usually develop, whether due to infections, long hospital stays and interaction with the hospital environment or increased antimicrobials consumption (Petersen and Round, 2014; Kim et al., 2017). It is noteworthy the high abundance of HAIrB found in PT rectum, especially *E. coli* and *K. pneumoniae*, a pattern also described in other studies (Ravi et al., 2019; Mahnic et al., 2020). In PT hands, in addition of *S. epidermidis*, *E. coli* and *A. baumannii* abundances stand out. We found *A. baumannii* oligotypes from PT mainly shared with bed equipment and HCW hands and protective clothing. Other studies also report the contamination of HCW hands and protective clothing after patient care, especially the transfer of *A. baumannii* (Morgan et al., 2012; Munoz-Price et al., 2012; Puig-Asensio et al., 2020). The different PT sites showed similar AMR patterns, but with higher positivity for rectum. As well as for PT sites, BL genes are also the same detected in HE and HCW, with different proportions of positivity. Despite HCW protective clothing and hands showing similar loads of HAIrB, the detection of BL genes showed greater positivity in the hands, reinforcing the possible insufficient adherence to hand hygiene protocols. Although studies have demonstrated HCW mobile phone contamination by HAIrB, in some cases with AMR (Bhardwaj et al., 2020), in the present study HCW mobile phones showed low HAIrB load and null or low AMR.

The agreement found between the CD and CI approaches, both in terms of bacterial identification and AMR profile, demonstrates the clinical relevance of CI approach findings (not just about “dead cells DNA” findings). Previous studies have also shown agreement between CD and CI methods, however, they emphasize that HTS-based CI methods are able to capture greater bacterial diversity, not restricted to specific groups, and that, even with their limitations, must be considered as important tools for investigation of bacterial communities in hospital environments and to HAI control (Oberauner et al., 2013; Comar et al., 2019). The CI approach also has the increment of providing a big picture, both by identifying the bacterial diversity closest to reality, as well as by screening AMR profiles, using leaner laboratory tests allied to robust bioinformatics pipelines (Boers et al., 2019; Chiu and Miller, 2019; Cruz et al., 2021).

This study has some limitations for routine implementations in hospitals. Although basic molecular biology techniques such as real-time PCR are currently available within major hospital’s laboratories in Brazil, DNA sequencing and downstream sequence analysis are not a reality, requiring specialized equipment and professional qualification. However, comparing the direct and indirect costs, the time and the precision of the output toward early and specific detection of HAI-related bacteria between traditional detection techniques and the herein described method, we can see important gains such as shorter hospital stays for patients, rational, appropriate and effective use of antimicrobials, adoption of preventive and controls measures concerning outbreaks. Despite having decreased overtime, direct costs for a DNA sequencing-monitoring of HAI-related bacteria are still higher if compared to conventional methods, but far less time consuming and much more accurate. Taken together, these new technologies also allow continuing education toward adoption of practices for patient and HCW safety, promoting a healthier hospital environment.

This study has assessed the HAI-related bacteria colonization profile of a developing country public hospital over time, occupants and space, demonstrating a variety and the spreading of HAIrB harboring several genes related to drug resistance in the hospital environment, among patients and healthcare workers. Being far more sensitive, informative and less time-consuming than traditional methods for assessment of AMR-HAIrB, DNA sequencing-based methods identified contamination hotspots and revealed that they seem to be constant over time. The scenario, as determined in this study, can be extrapolated to other hospitals of the public health system of developing countries, allowing revision of medical, nursery and general practices applied to the HAI control. In conclusion, active surveillance programs based on microbiome follow-up are useful for the assessment and control of HAI, indicating the needs of a multidisciplinary approach.

## Data Availability Statement

The datasets presented in this study can be found in online repositories. The names of the repository/repositories and accession number(s) can be found below: https://www.ncbi.nlm.nih.gov/, BioProject PRJNA728360.

## Ethics Statement

This study involves human participants and was reviewed and approved by the Research Ethics Committee of Federal University of Santa Catarina (Number 32930514.0.0000.0121). The patients/participants provided their written informed consent to participate in this study.

## Author Contributions

AS, LO, TS, and EG: conceptualization, project administration, and supervision. AS, AC, GNC, GCC, PC, DT, CZ, and LO: data curation and formal analysis. AS, GNC, PC, DT, CZ, TK, IM, CS, MV, and MS: investigation and methodology. AS and GNC: validation. AS: writing—original draft. AS, AC, LO, TS, and EG: writing—review and editing. All authors contributed to the article and approved the submitted version.

## Conflict of Interest

 AS, AC, GNC, GCC, and LO are currently full-time employees of BiomeHub (SC, Brazil), a research and consulting company specialized in microbiome technologies. This study received funding from BiomeHub (SC, Brazil). The funder had the following involvement with the study: supply of reagents, consumables, infrastructure and equipment for analysis. The remaining authors declare that the research was conducted in the absence of any commercial or financial relationships that could be construed as a potential conflict of interest.

## Publisher’s Note

All claims expressed in this article are solely those of the authors and do not necessarily represent those of their affiliated organizations, or those of the publisher, the editors and the reviewers. Any product that may be evaluated in this article, or claim that may be made by its manufacturer, is not guaranteed or endorsed by the publisher.

## Supplementary Material

Supplementary Figure 1 Experimental design of Healthcare-associated Infections Microbiome Project (HAIMP).

Supplementary Figure 2 Oligotypes tracking in culture-independent and culture-dependent analyses.

Supplementary Figure 3 Risk map of Intensive Care Unit of a tertiary public hospital along 6-month surveillance program with 16S rRNA gene sequencing method.

Supplementary Figure 4 Risk map of Internal Medicine Ward of a tertiary public hospital along 6-month surveillance program with 16S rRNA gene sequencing method.

Supplementary Figure 5 Risk map of Surgical Ward of a tertiary public hospital along 6-month surveillance program with 16S rRNA gene sequencing method.

Supplementary Figure 6 Risk map of Emergency of a tertiary public hospital along 6-month surveillance program with 16S rRNA gene sequencing method.

Supplementary Figure 7 Risk map of General Surgery Unit of a tertiary public hospital along 6-month surveillance program with 16S rRNA gene sequencing method.

Supplementary Figure 8 Agreement analysis of taxonomic classification between Vitek^®^ 2 and 16S rRNA gene sequencing.

Supplementary Figure 9 Identification of beta-lactamases genes in antimicrobial-resistant Gram-negative bacteria isolates.

Supplementary Table 1 List of the sampling points collected in duplicate in a tertiary teaching hospital for culture-dependent (CD) and culture-independent (CI) methods.

Supplementary Table 2 List of HAIrB expanded group.
