# Supplementary figures and images for: Healthcare-Associated Infections-Related Bacteriome and Antimicrobial Resistance Profiling: Assessing Contamination Hotspots in a Developing Country Public Hospital
Source: Front Microbiol. 2021 Aug 16;12:711471. doi: 10.3389/fmicb.2021.711471 (PMC8415557; doi:10.3389/fmicb.2021.711471)

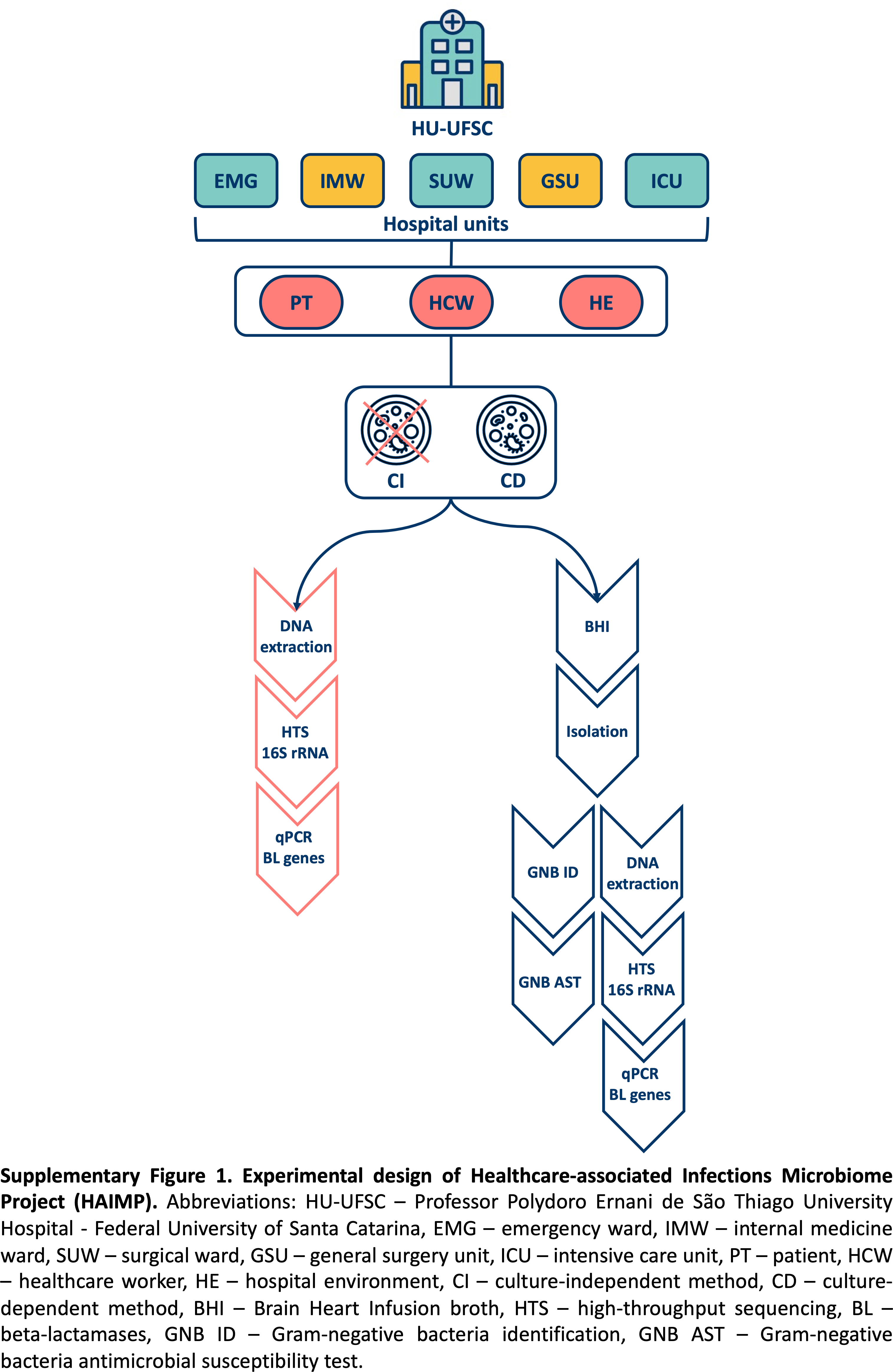

Supplement: Supplementary Figure 1 — Experimental design of Healthcare-associated Infections Microbiome Project (HAIMP). [file Image_1.TIFF]

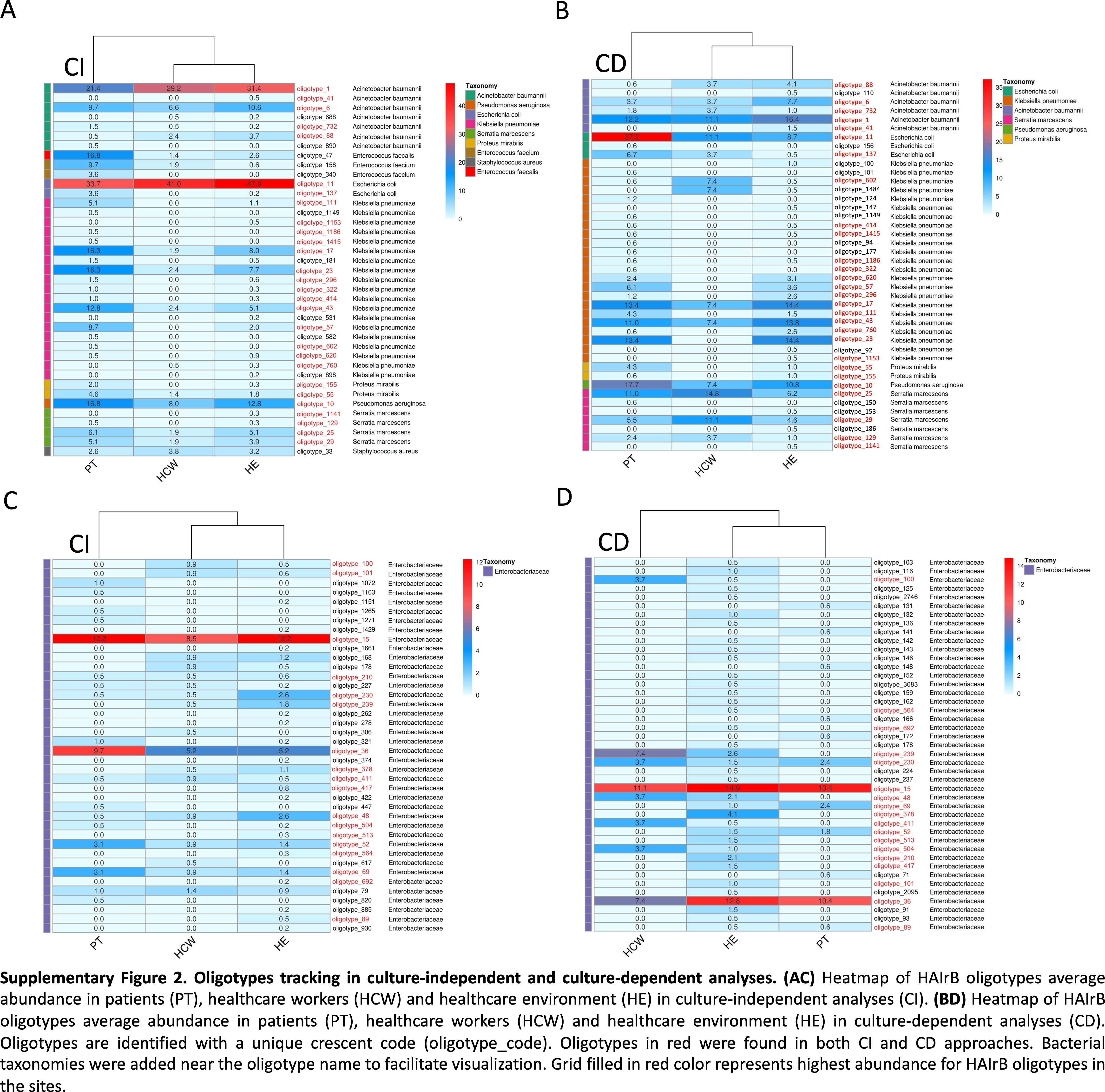

Supplement: Supplementary Figure 2 — Oligotypes tracking in culture-independent and culture-dependent analyses. [file Image_2.TIFF]

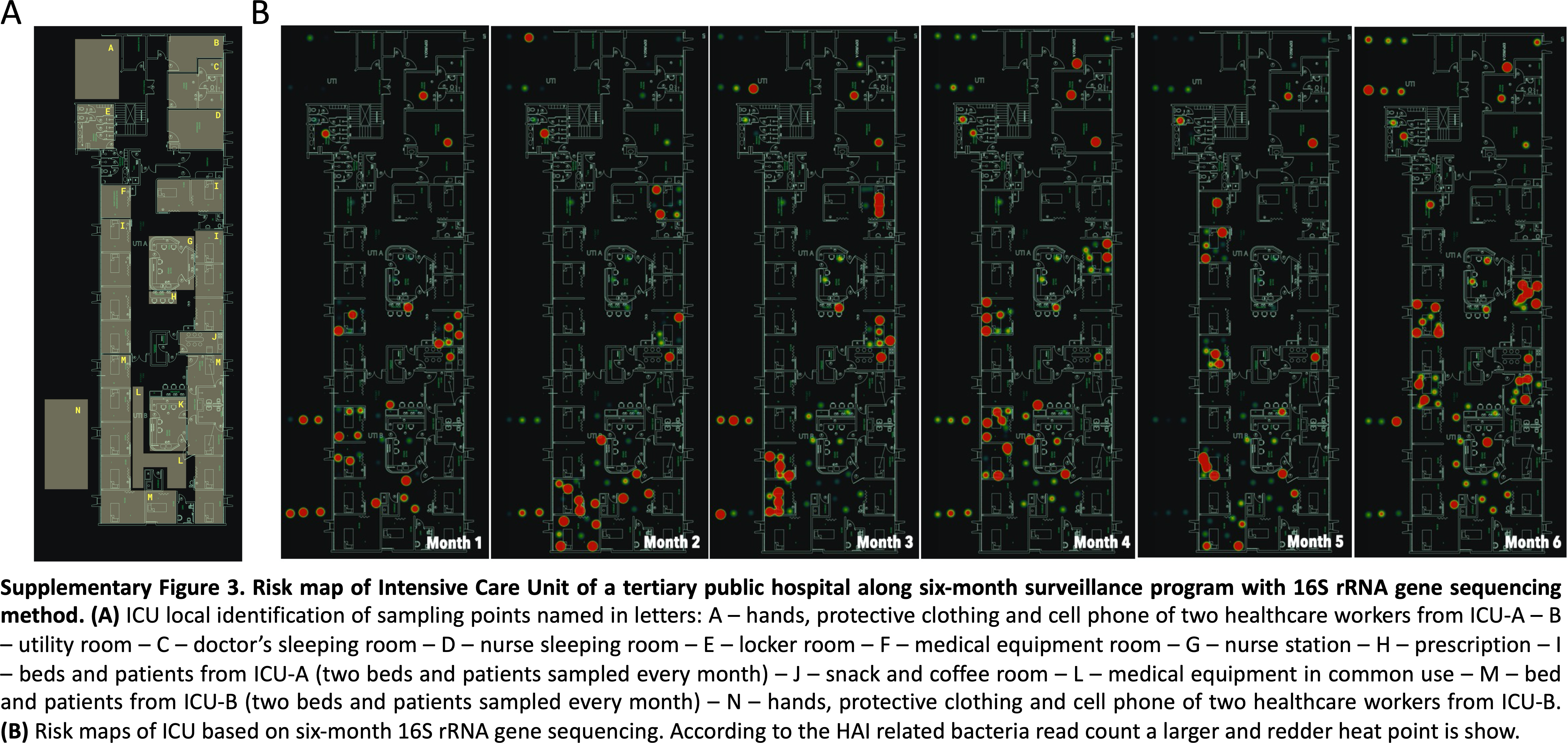

Supplement: Supplementary Figure 3 — Risk map of Intensive Care Unit of a tertiary public hospital along 6-month surveillance program with 16S rRNA gene sequencing method. [file Image_3.TIFF]

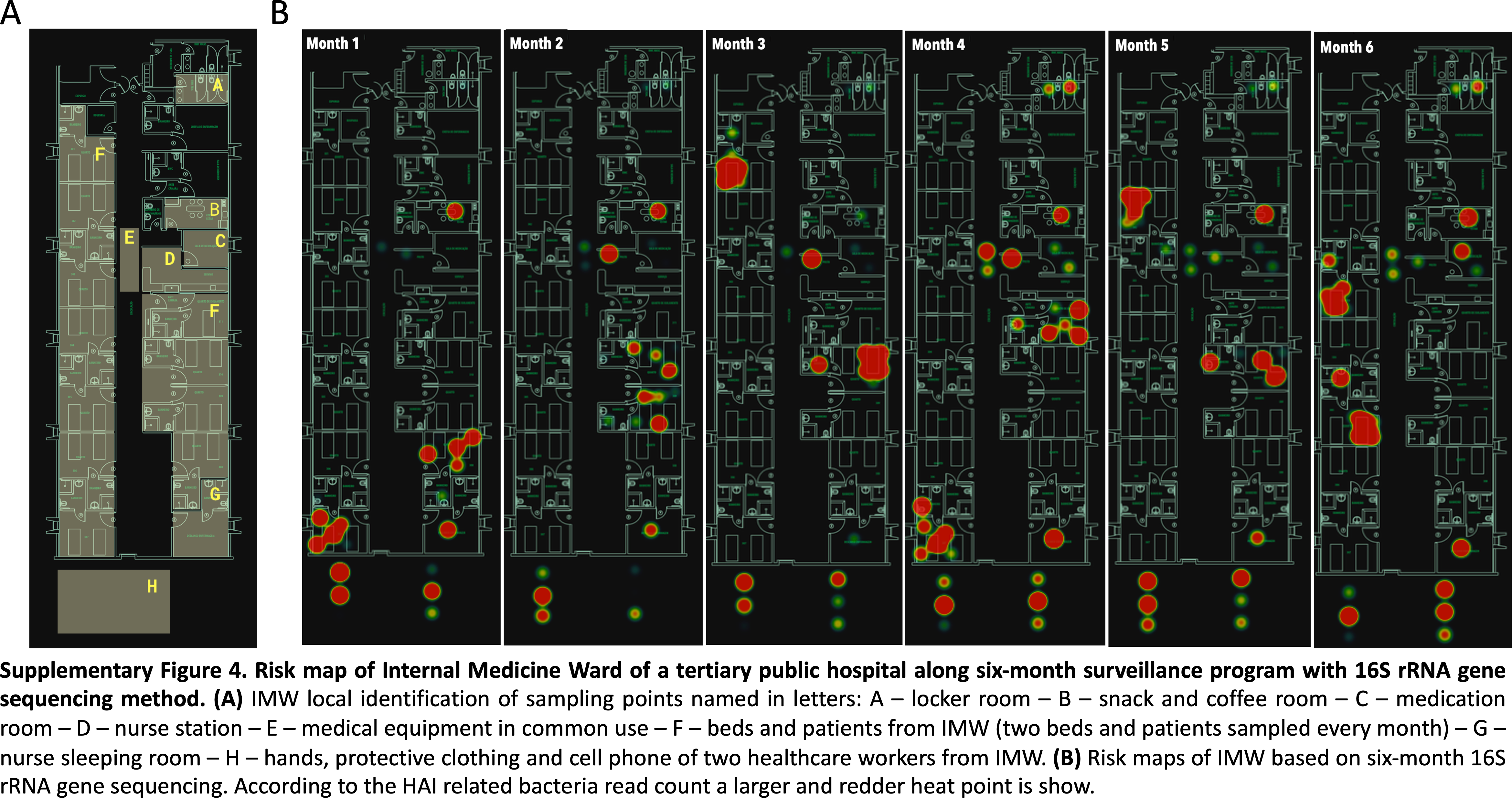

Supplement: Supplementary Figure 4 — Risk map of Internal Medicine Ward of a tertiary public hospital along 6-month surveillance program with 16S rRNA gene sequencing method. [file Image_4.TIFF]

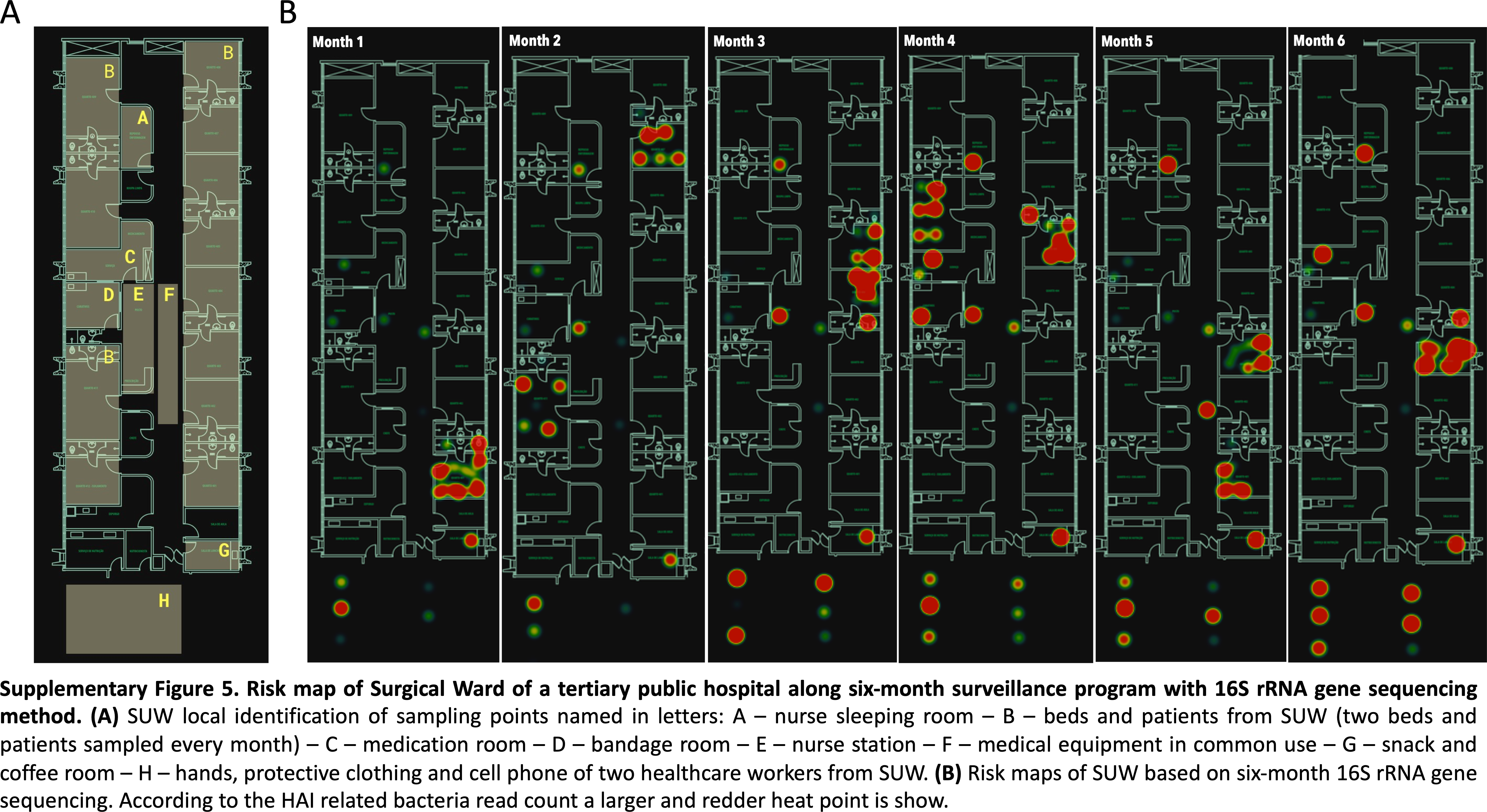

Supplement: Supplementary Figure 5 — Risk map of Surgical Ward of a tertiary public hospital along 6-month surveillance program with 16S rRNA gene sequencing method. [file Image_5.TIFF]

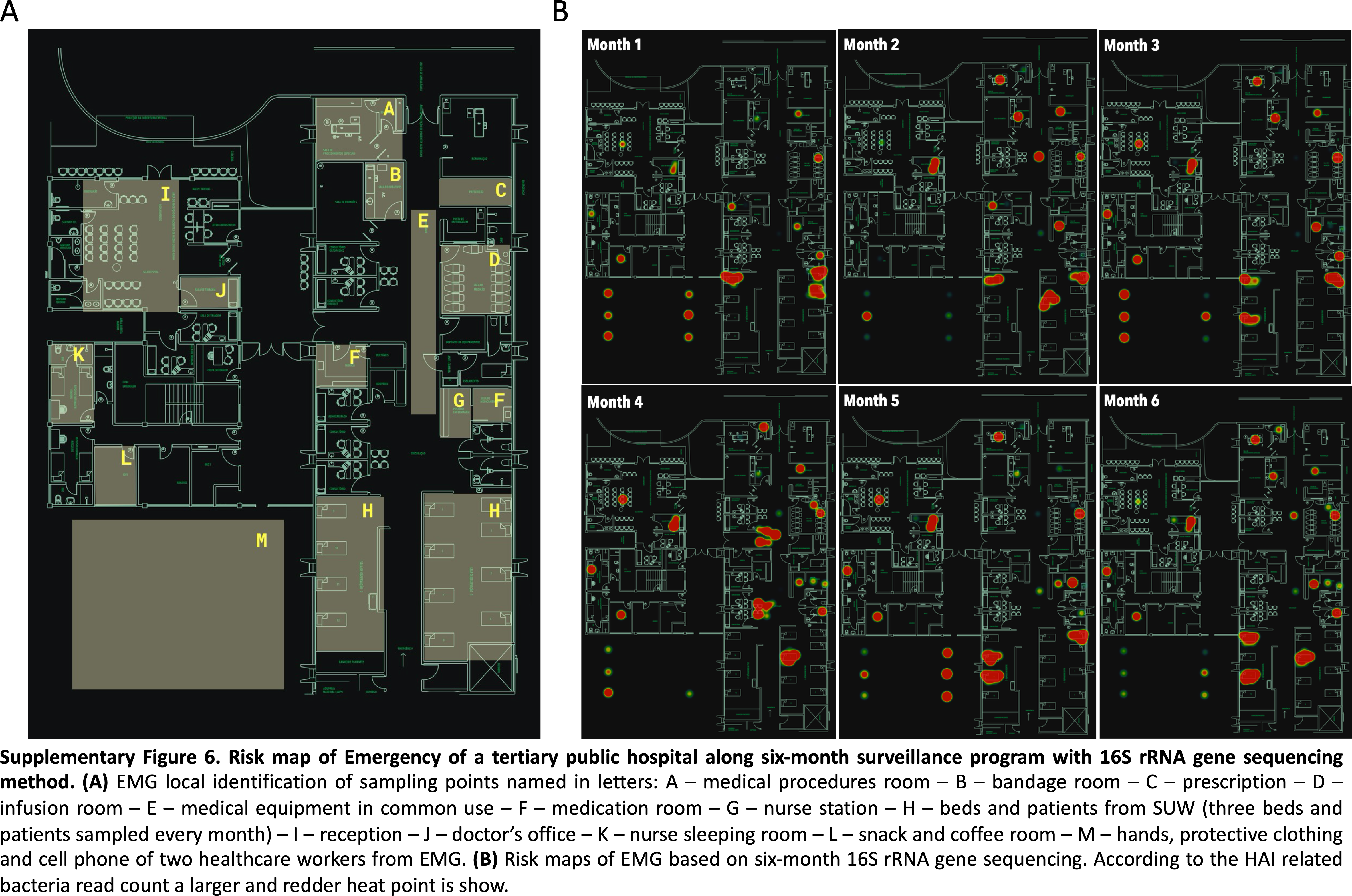

Supplement: Supplementary Figure 6 — Risk map of Emergency of a tertiary public hospital along 6-month surveillance program with 16S rRNA gene sequencing method. [file Image_6.TIFF]

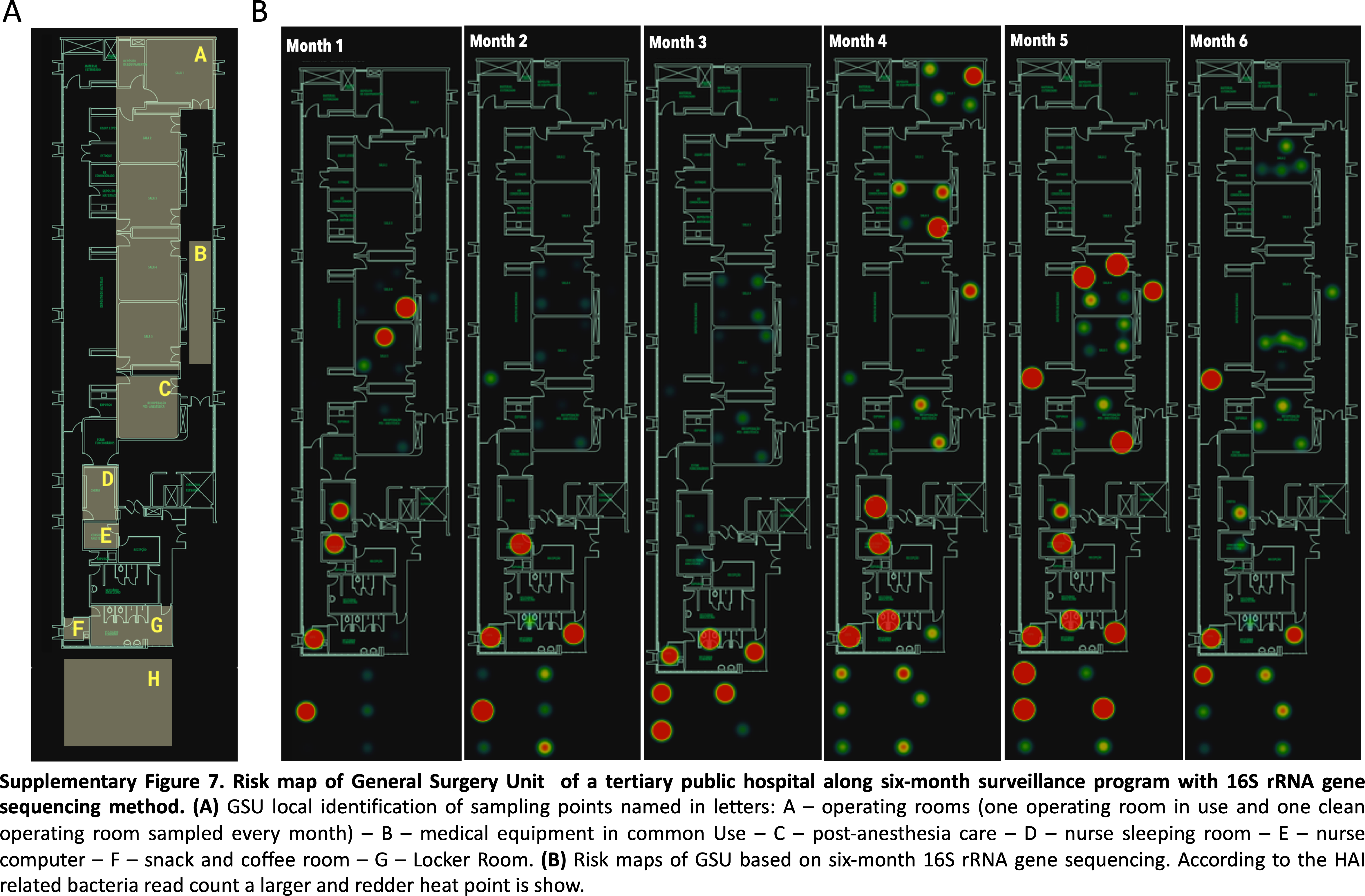

Supplement: Supplementary Figure 7 — Risk map of General Surgery Unit of a tertiary public hospital along 6-month surveillance program with 16S rRNA gene sequencing method. [file Image_7.TIFF]

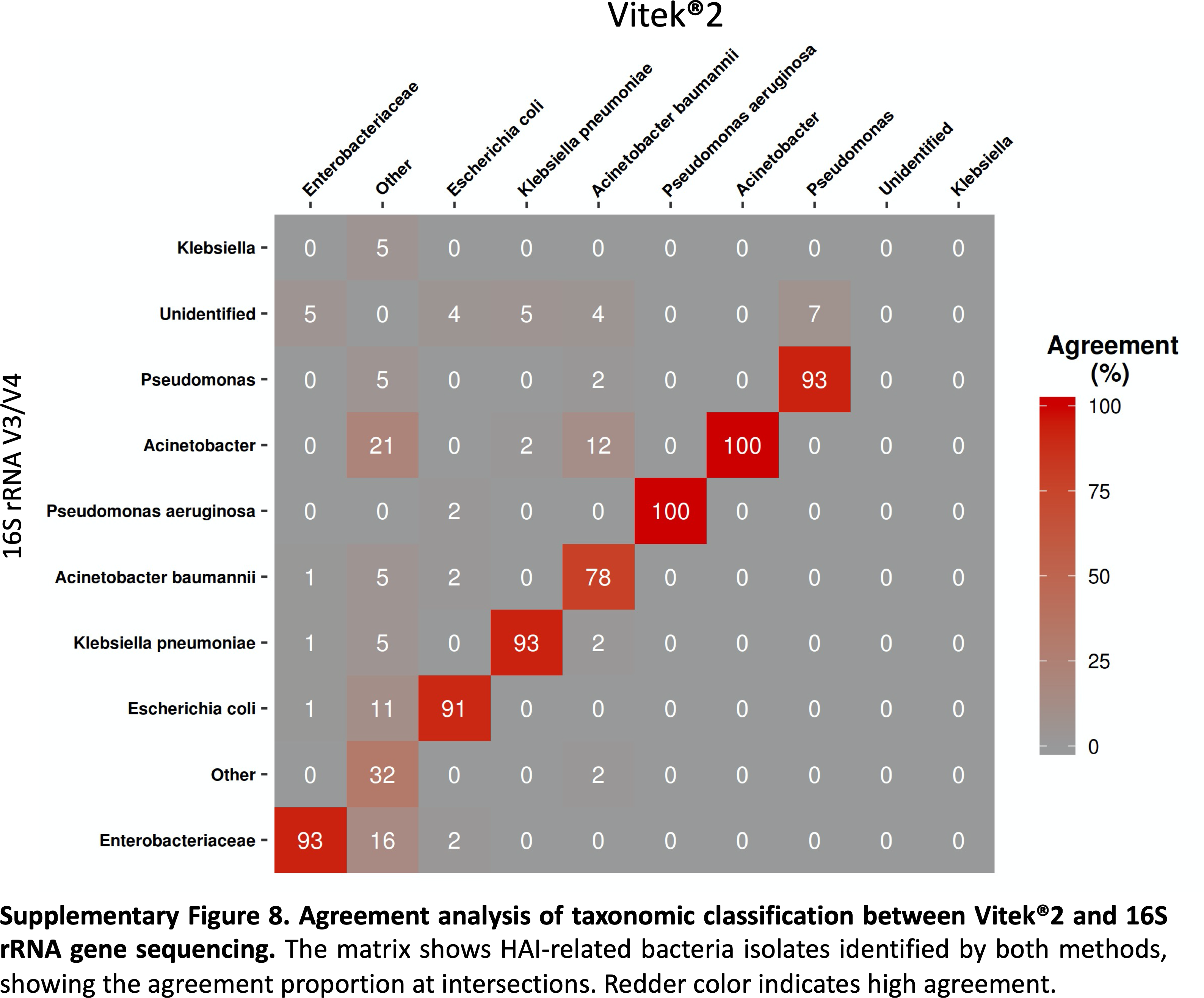

Supplement: Supplementary Figure 8 — Agreement analysis of taxonomic classification between Vitek® 2 and 16S rRNA gene sequencing. [file Image_8.TIFF]

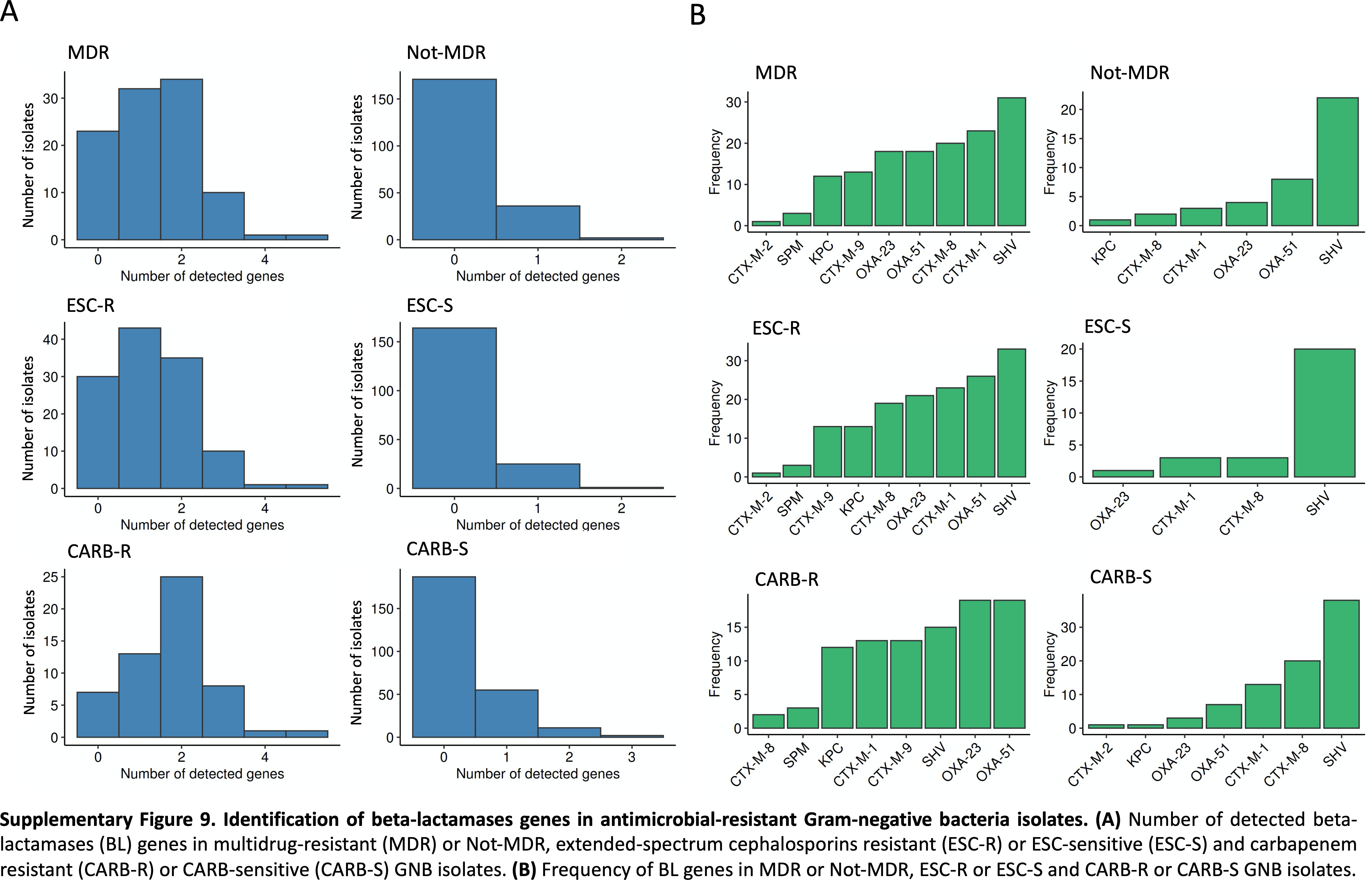

Supplement: Supplementary Figure 9 — Identification of beta-lactamases genes in antimicrobial-resistant Gram-negative bacteria isolates. [file Image_9.TIFF]
