# Supplementary material for: Healthcare-Associated Infections-Related Bacteriome and Antimicrobial Resistance Profiling: Assessing Contamination Hotspots in a Developing Country Public Hospital
Source: Front Microbiol. 2021 Aug 16;12:711471. doi: 10.3389/fmicb.2021.711471 (PMC8415557; doi:10.3389/fmicb.2021.711471)
Supplement: Supplementary Table 1 — List of the sampling points collected in duplicate in a tertiary teaching hospital for culture-dependent (CD) and culture-independent (CI) methods. [file Table_1.DOCX]

Supplementary Table 1. List of the sampling points collected in duplicate in a tertiary teaching hospital for culture-dependent (CD) and culture-independent (CI) methods

| Sampling point N° | Unit | Sample | Description |
| --- | --- | --- | --- |
| 1 | EMG | HCW-1 | Hands |
| 2 | EMG | HCW-1 | Protective Clothing (White Coat or Scrub) |
| 3 | EMG | HCW-1 | Mobile Phone |
| 4 | EMG | HCW-2 | Hands |
| 5 | EMG | HCW-2 | Protective Clothing (White Coat or Scrub) |
| 6 | EMG | HCW-2 | Mobile Phone |
| 7 | EMG | PT-1 | Rectum |
| 8 | EMG | PT-1 | Hands |
| 9 | EMG | PT-1 | Nasal |
| 10 | EMG | PT-2 | Rectum |
| 11 | EMG | PT-2 | Hands |
| 12 | EMG | PT-2 | Nasal |
| 13 | EMG | PT-3 | Rectum |
| 14 | EMG | PT-3 | Hands |
| 15 | EMG | PT-3 | Nasal |
| 16 | EMG | Bed 1 | Bed Rail |
| 17 | EMG | Bed 1 | Medical Gas Regulator and IV Stand |
| 18 | EMG | Bed 2 | Bed Rail |
| 19 | EMG | Bed 2 | Medical Gas Regulator and IV Stand |
| 20 | EMG | Bed 3 | Bed Rail |
| 21 | EMG | Bed 3 | Medical Gas Regulator and IV Stand |
| 22 | EMG | HCW Bathroom | Soap Dispenser, Tap and Flush Button |
| 23 | EMG | Nurse Station | Workstation, Computer Keyboard and Telephone |
| 24 | EMG | Medication Room | Soap Dispenser and Tap |
| 25 | EMG | Medication Room | High Touch Surfaces |
| 26 | EMG | Infusion Room | Patient Chair |
| 27 | EMG | Infusion Room | IV Stand |
| 28 | EMG | Prescription | Computers |
| 29 | EMG | Medical Procedures Room | Scrub Sink |
| 30 | EMG | Medical Procedures Room | Bed |
| 31 | EMG | Bandage Room | High Touch Surfaces |
| 32 | EMG | Doctor’s office | Bed |
| 33 | EMG | Doctor’s office | Computer Desk |
| 34 | EMG | Doctor’s office | Soap Dispenser and Tap |
| 35 | EMG | Reception | Reception Chairs |
| 36 | EMG | HCW Snack and Coffee Room | High Touch Surfaces |
| 37 | EMG | Nurse Sleeping Room | High Touch Surfaces |
| 38 | EMG | Hands Hygiene Device | Alcohol Gel Dispenser |
| 39 | EMG | Medical Equipment of Common Use | Medication Cart |
| 40 | IMW | HCW-1 | Hands |
| 41 | IMW | HCW-1 | Protective Clothing (White Coat or Scrub) |
| 42 | IMW | HCW-1 | Mobile Phone |
| 43 | IMW | HCW-2 | Hands |
| 44 | IMW | HCW-2 | Protective Clothing (White Coat or Scrub) |
| 45 | IMW | HCW-2 | Mobile Phone |
| 46 | IMW | PT-1 | Rectum |
| 47 | IMW | PT-1 | Hands |
| 48 | IMW | PT-1 | Nasal |
| 49 | IMW | PT-2 | Rectum |
| 50 | IMW | PT-2 | Hands |
| 51 | IMW | PT-2 | Nasal |
| 52 | IMW | Bed 1 | Soap Dispenser, Tap and Flush Button of the Bathroom |
| 53 | IMW | Bed 1 | Bed Rail |
| 54 | IMW | Bed 1 | Medical Gas Regulator and IV Stand |
| 55 | IMW | Bed 2 | Soap Dispenser, Tap and Flush Button of the Bathroom |
| 56 | IMW | Bed 2 | Bed Rail |
| 57 | IMW | Bed 2 | Medical Gas Regulator and IV Stand |
| 58 | IMW | Nurse Station | Workstation, Computer Keyboard and Telephone |
| 59 | IMW | Medication Room | Soap Dispenser and Tap |
| 60 | IMW | Medication Room | High Touch Surfaces |
| 61 | IMW | Locker Room | Lockers |
| 62 | IMW | Locker Room | Soap Dispenser, Tap and Flush Button of the Bathroom |
| 63 | IMW | HCW Snack and Coffee Room | High Touch Surfaces |
| 64 | IMW | Nurse Sleeping Room | High Touch Surfaces |
| 65 | IMW | Hands Hygiene Device | Alcohol Gel Dispenser |
| 66 | IMW | Medical Equipment of Common Use | Portable Glucose Analyzer |
| 67 | SUW | HCW-1 | Hands |
| 68 | SUW | HCW-1 | Protective Clothing (White Coat or Scrub) |
| 69 | SUW | HCW-1 | Mobile Phone |
| 70 | SUW | HCW-2 | Hands |
| 71 | SUW | HCW-2 | Protective Clothing (White Coat or Scrub) |
| 72 | SUW | HCW-2 | Mobile Phone |
| 73 | SUW | PT-1 | Rectum |
| 74 | SUW | PT-1 | Hands |
| 75 | SUW | PT-1 | Nasal |
| 76 | SUW | PT-2 | Rectum |
| 77 | SUW | PT-2 | Hands |
| 78 | SUW | PT-2 | Nasal |
| 79 | SUW | Bed 1 | Soap Dispenser, Tap and Flush Button of the Bathroom |
| 80 | SUW | Bed 1 | Medical Gas Regulator and IV Stand |
| 81 | SUW | Bed 1 | Bed Rail |
| 82 | SUW | Bed 2 | Soap Dispenser, Tap and Flush Button of the Bathroom |
| 83 | SUW | Bed 2 | Bed Rail |
| 84 | SUW | Bed 2 | Medical Gas Regulator and IV Stand |
| 85 | SUW | Nurse Station | Workstation, Computer Keyboard and Telephone |
| 86 | SUW | Medication Room | Soap Dispenser and Tap |
| 87 | SUW | Medication Room | High Touch Surfaces |
| 88 | SUW | Bandage Room | High Touch Surfaces |
| 89 | SUW | HCW Snack and Coffee Room | High Touch Surfaces |
| 90 | SUW | Nurse Sleeping Room | High Touch Surfaces |
| 91 | SUW | Hands Hygiene Device | Alcohol Gel Dispenser |
| 92 | SUW | Medical Equipment of Common Use | Portable Glucose Analyzer |
| 93 | GSU | HCW-1 | Hands |
| 94 | GSU | HCW-1 | Protective Clothing (White Coat or Scrub) |
| 95 | GSU | HCW-1 | Mobile Phone |
| 96 | GSU | HCW-2 | Hands |
| 97 | GSU | HCW-2 | Protective Clothing (White Coat or Scrub) |
| 98 | GSU | HCW-2 | Mobile Phone |
| 99 | GSU | Post-anesthesia Care | Bed Rail |
| 100 | GSU | Post-anesthesia Care | Medical Equipment |
| 101 | GSU | Post-anesthesia Care | High Touch Surfaces |
| 102 | GSU | Operating Room in Use | Surgical Table |
| 103 | GSU | Operating Room in Use | Anesthesia Cart - High Touch Surfaces |
| 104 | GSU | Operating Room in Use | Laryngoscope Tray |
| 105 | GSU | Operating Room in Use | Injectable Anesthetics Tray |
| 106 | GSU | Clean Operating Room | Surgical Table |
| 107 | GSU | Clean Operating Room | Surgical Light |
| 108 | GSU | Clean Operating Room | Infusion Pump |
| 109 | GSU | Clean Operating Room | Electrosurgical Generator |
| 110 | GSU | Locker Room | Lockers |
| 111 | GSU | Locker Room | Soap Dispenser and Tap |
| 112 | GSU | Nurse Sleeping Room | High Touch Surfaces |
| 113 | GSU | HCW Snack and Coffee Room | High Touch Surfaces |
| 114 | GSU | Medical Equipment of Common Use | Nurse Computer |
| 115 | GSU | Medical Equipment of Common Use | Stretcher |
| 116 | GSU | Hands Hygiene Device | Alcohol Gel Dispenser |
| 117 | ICU-A | HCW-1 | Hands |
| 118 | ICU-A | HCW-1 | Protective Clothing (White Coat or Scrub) |
| 119 | ICU-A | HCW-1 | Mobile Phone |
| 120 | ICU-A | HCW-2 | Hands |
| 121 | ICU-A | HCW-2 | Protective Clothing (White Coat or Scrub) |
| 122 | ICU-A | HCW-2 | Mobile Phone |
| 123 | ICU-A | PT-1 | Rectum |
| 124 | ICU-A | PT-1 | Hands |
| 125 | ICU-A | PT-1 | Nasal |
| 126 | ICU-A | PT-2 | Rectum |
| 127 | ICU-A | PT-2 | Hands |
| 128 | ICU-A | PT-2 | Nasal |
| 129 | ICU-A | Bed 1 | Bed Rail |
| 130 | ICU-A | Bed 1 | Infusion Pump |
| 131 | ICU-A | Bed 1 | Heart Monitor |
| 132 | ICU-A | Bed 1 | Curtain |
| 133 | ICU-A | Bed 2 | Bed Rail |
| 134 | ICU-A | Bed 2 | Utility Cart |
| 135 | ICU-A | Bed 2 | Supporting Wall of Personal Protective Clothing |
| 136 | ICU-A | Bed 2 | Thermometer |
| 137 | ICU-A | Nurse Station | Workstation, Computer Keyboard and Telephone |
| 138 | ICU-A | Nurse Station | Medical Records Clipboard |
| 139 | ICU-A | Prescription | Computer Keyboard |
| 140 | ICU-A | Medical Equipment Room | Clean Medical Equipment |
| 141 | ICU-A | Locker Room | Lockers |
| 142 | ICU-A | Locker Room | Soap Dispenser, Tap and Flush Button of the Bathroom |
| 143 | ICU-A | Utility Room | High Touch Surfaces |
| 144 | ICU-A | Utility Room | Dryer |
| 145 | ICU-A | Doctor’s Sleeping Room | High Touch Surfaces |
| 146 | ICU-A | Nurse Sleeping Room | High Touch Surfaces |
| 147 | ICU-A | HCW Snack and Coffee Room | High Touch Surfaces |
| 148 | ICU-B | HCW-1 | Hands |
| 149 | ICU-B | HCW-1 | Protective Clothing (White Coat or Scrub) |
| 150 | ICU-B | HCW-1 | Mobile Phone |
| 151 | ICU-B | HCW-2 | Hands |
| 152 | ICU-B | HCW-2 | Protective Clothing (White Coat or Scrub) |
| 153 | ICU-B | HCW-2 | Mobile Phone |
| 154 | ICU-B | PT-1 | Rectum |
| 155 | ICU-B | PT-1 | Hands |
| 156 | ICU-B | PT-1 | Nasal |
| 157 | ICU-B | PT-2 | Rectum |
| 158 | ICU-B | PT-2 | Hands |
| 159 | ICU-B | PT-2 | Nasal |
| 160 | ICU-B | Bed 1 | Bed Rail |
| 161 | ICU-B | Bed 1 | Infusion Pump |
| 162 | ICU-B | Bed 1 | Heart Monitor |
| 163 | ICU-B | Bed 1 | Curtain |
| 164 | ICU-B | Bed 2 | Bed Rail |
| 165 | ICU-B | Bed 2 | Utility Cart |
| 166 | ICU-B | Bed 2 | Supporting Wall of Personal Protective Clothing |
| 167 | ICU-B | Bed 2 | Thermometer |
| 168 | ICU-B | Nurse Station | Workstation, Computer Keyboard and Telephone |
| 169 | ICU-B | Nurse Station | Refrigerator |
| 170 | ICU-B | Prescription | Computer Keyboard |
| 171 | ICU-B | Hands Hygiene Device | Alcohol Gel Dispenser |
| 172 | ICU-B | Medical Equipment of Common Use | Clean Clothes Cart |
| 173 | ICU-B | Medical Equipment of Common Use | Portable Glucose Analyzer |
| 174 | ICU-B | Medical Equipment of Common Use | Cuff Pressure Gauge |
| 175 | ICU-B | Medical Equipment of Common Use | Portable Shower Bath |
| 176 | ICU-B | Medical Equipment of Common Use | Crash Cart |
| 177 | ICU-B | Medical Equipment of Common Use | Portable Image Intensifier (X-Ray) |
| 178 | ICU-B | Medical Equipment of Common Use | Ultrasound Machine |
| 179 | ICU-B | Medical Equipment of Common Use | Patient Transfer Board |
| 180 | ICU-B | Medical Equipment of Common Use | Physiotherapy Chair |

HCW — healthcare worker, PT — patient, EMG — emergency ward, IMW — internal medicine ward, SUW — surgical ward, GSU — general surgery unit, ICU-A and ICU-B — intensive care unit A and B.
