# Supplementary material for: Healthcare-Associated Infections-Related Bacteriome and Antimicrobial Resistance Profiling: Assessing Contamination Hotspots in a Developing Country Public Hospital
Source: Front Microbiol. 2021 Aug 16;12:711471. doi: 10.3389/fmicb.2021.711471 (PMC8415557; doi:10.3389/fmicb.2021.711471)
Supplement: Supplementary Table 2 — List of HAIrB expanded group. [file Table_2.DOCX]

**Supplementary Table 2.** List of HAIrB expanded group

| Species | Species | Species |
| --- | --- | --- |
| *Abiotrophia defective* | *Enterococcus durans* | *Mycoplasma penetrans* |
| *Achromobacter denitrificans* | *Enterococcus faecalis* | *Mycoplasma pneumoniae* |
| *Achromobacter xylosoxidans* | *Enterococcus faecium* | *Neisseria animaloris* |
| *Acinetobacter baumannii* | *Enterococcus gallinarum* | *Neisseria gonorrhoeae* |
| *Acinetobacter baylyi* | *Enterococcus hirae* | *Neisseria meningitidis* |
| *Acinetobacter bereziniae* | *Enterococcus raffinosus* | *Neisseria zoodegmatis* |
| *Acinetobacter calcoaceticus* | *Enterococcus saccharolyticus* | *Ochrobactrum anthropi* |
| *Acinetobacter gyllenbergii* | *Erysipelothrix rhusiopathiae* | *Oligella ureolytica* |
| *Acinetobacter haemolyticus* | *Escherichia albertii* | *Pantoea agglomerans* |
| *Acinetobacter indicus* | *Escherichia coli* | *Pantoea ananatis* |
| *Acinetobacter johnsonii* | *Escherichia fergusonii* | *Pantoea dispersa* |
| *Acinetobacter junii* | *Facklamia hominis* | *Pantoea septica* |
| *Acinetobacter lwoffii* | *Francisella tularensis* | *Pantoea stewartii* |
| *Acinetobacter nosocomialis* | *Fusobacterium nucleatum* | *Paracoccus yeei* |
| *Acinetobacter parvus* | *Gardnerella vaginalis* | *Pasteurella aerogenes* |
| *Acinetobacter pittii* | *Gemella bergeri* | *Pasteurella canis* |
| *Acinetobacter radioresistens* | *Gemella haemolysans* | *Pasteurella dagmatis* |
| *Acinetobacter schindleri* | *Gemella morbillorum* | *Pasteurella multocida* |
| *Acinetobacter soli* | *Gemella sanguinis* | *Pasteurella pneumotropica* |
| *Acinetobacter towneri* | *Globicatella sanguinis* | *Pasteurella testudinis* |
| *Acinetobacter ursingii* | *Globicatella sulfidifaciens* | *Pediococcus acidilactici* |
| *Actinobacillus ureae* | *Granulicatella adiacens* | *Shigella flexneri* |
| *Actinomyces israelii* | *Granulicatella elegans* | *Shigella sonnei* |
| *Aerococcus urinae* | *Grimontia hollisae* | *Sphingobacterium multivorum* |
| *Aerococcus viridans* | *Haemophilus influenzae* | *Sphingobacterium spiritivorum* |
| *Aeromonas caviae* | *Haemophilus parainfluenzae* | *Sphingobacterium thalpophilum* |
| *Aeromonas hydrophila* | *Helcococcus kunzii* | *Sphingomonas paucimobilis* |
| *Aeromonas salmonicida* | *Helicobacter pylori* | *Staphylococcus arlettae* |
| *Aeromonas sobria* | *Klebsiella* spp. | *Staphylococcus aureus* |
| *Aeromonas veronii* | *Kluyvera ascorbata* | *Staphylococcus auricularis* |
| *Alloiococcus otitis* | *Kytococcus sedentarius* | *Staphylococcus capitis* |
| *Anaplasma phagocytophilum* | *Lactococcus garvieae* | *Staphylococcus caprae* |
| *Bacillus anthracis* | *Lactococcus raffinolactis* | *Staphylococcus chromogenes* |
| *Bacillus cereus* | *Leclercia adecarboxylata* | *Staphylococcus cohnii* |
| *Bacillus subtilis* | *Legionella pneumophila* | *Staphylococcus epidermidis* |
| *Bacteroides fragilis* | *Leptospira interrogans* | *Staphylococcus equorum* |
| *Bartonella henselae* | *Leuconostoc citreum* | *Staphylococcus gallinarum* |
| *Bartonella quintana* | *Leuconostoc mesenteroides* | *Staphylococcus haemolyticus* |
| *Bordetella bronchiseptica* | *Leuconostoc pseudomesenteroides* | *Staphylococcus hominis* |
| *Bordetella hinzii* | *Listeria grayi* | *Staphylococcus hyicus* |
| *Bordetella pertussis* | *Listeria innocua* | *Staphylococcus intermedius* |
| *Bordetella trematum* | *Listeria ivanovii* | *Staphylococcus kloosii* |
| *Borrelia burgdorferi* | *Listeria monocytogenes* | *Staphylococcus lentus* |
| *Cronobacter sakazakii* | *Listeria seeligeri* | *Staphylococcus lugdunensis* |
| *Cupriavidus pauculus* | *Listeria welshimeri* | *Staphylococcus pettenkoferi* |
| *Delftia acidovorans* | *Mannheimia haemolytica* | *Staphylococcus pseudintermedius* |
| *Dermacoccus nishinomiyaensis* | *Micrococcus luteus* | *Staphylococcus saprophyticus* |
| *Edwardsiella hoshinae* | *Micrococcus lylae* | *Staphylococcus schleiferi* |
| *Edwardsiella tarda* | *Moellerella wisconsensis* | *Staphylococcus sciuri* |
| *Ehrlichia chaffeensis* | *Moraxella catarrhalis* | *Staphylococcus simulans* |
| *Elizabethkingia meningoseptica* | *Mycobacterium avium* | *Staphylococcus vitulinus* |
| *Enterobacter* spp. | *Mycobacterium intracellulare* | *Staphylococcus warneri* |
| *Enterococcus avium* | *Mycobacterium leprae* | *Staphylococcus xylosus* |
| *Enterococcus casseliflavus* | *Mycobacterium tuberculosis* | *Stenotrophomonas maltophilia* |
| *Enterococcus cecorum* | *Mycobacterium ulcerans* | *Streptococcus agalactiae* |
| *Enterococcus columbae* | *Mycoplasma hominis* | *Streptococcus alactolyticus* |
| *Streptococcus anginosus* | *Streptococcus porcinus* | *Vibrio parahaemolyticus* |
| *Streptococcus canis* | *Streptococcus pseudoporcinus* | *Vibrio vulnificus* |
| *Streptococcus cristatus* | *Streptococcus pyogenes* | *Yersinia aldovae* |
| *Streptococcus downei* | *Streptococcus salivarius* | *Yersinia enterocolitica* |
| *Streptococcus equinus* | *Streptococcus sanguinis* | *Yersinia frederiksenii* |
| *Streptococcus gordonii* | *Streptococcus sobrinus* | *Yersinia intermedia* |
| *Streptococcus hyointestinalis* | *Streptococcus suis* | *Yersinia kristensenii* |
| *Streptococcus infantarius* | *Streptococcus thoraltensis* | *Yersinia pestis* |
| *Streptococcus intermedius* | *Streptococcus uberis* | *Yersinia pseudotuberculosis* |
| *Streptococcus massiliensis* | *Streptococcus vestibularis* | *Yersinia ruckeri* |
| *Streptococcus mitis* | *Treponema denticola* | *Yokenella regensburgei* |
| *Streptococcus mutans* | *Treponema pallidum* |  |
| *Streptococcus oralis* | *Vagococcus fluvialis* |  |
| *Streptococcus ovis* | *Vibrio alginolyticus* |  |
| *Streptococcus parasanguinis* | *Vibrio cholerae* |  |
| *Streptococcus pasteurianus* | *Vibrio fluvialis* |  |
| *Streptococcus pluranimalium* | *Vibrio metschnikovii* |  |
| *Streptococcus pneumoniae* | *Vibrio mimicus* |  |
| *Streptococcus porcinus* | *Vibrio parahaemolyticus* |  |
| *Streptococcus pseudoporcinus* | *Vibrio vulnificus* |  |
| *Streptococcus pyogenes* | *Yersinia aldovae* |  |
| *Streptococcus salivarius* | *Yersinia enterocolitica* |  |
| *Streptococcus sanguinis* | *Yersinia frederiksenii* |  |
| *Streptococcus sobrinus* | *Yersinia intermedia* |  |
| *Streptococcus suis* | *Yersinia kristensenii* |  |
| *Streptococcus thoraltensis* | *Yersinia pestis* |  |
| *Streptococcus uberis* | *Yersinia pseudotuberculosis* |  |
| *Streptococcus vestibularis* | *Yersinia ruckeri* |  |
| *Treponema denticola* | *Yokenella regensburgei* |  |
| *Treponema pallidum* | *Streptococcus massiliensis* |  |
| *Vagococcus fluvialis* | *Streptococcus mitis* |  |
| *Vibrio alginolyticus* | *Streptococcus mutans* |  |
| *Vibrio cholerae* | *Streptococcus oralis* |  |
| *Vibrio fluvialis* | *Streptococcus ovis* |  |
| *Vibrio metschnikovii* | *Streptococcus parasanguinis* |  |
| *Vibrio mimicus* | *Streptococcus pasteurianus* |  |
| *Vibrio parahaemolyticus* | *Streptococcus pluranimalium* |  |
| *Vibrio vulnificus* | *Streptococcus pneumoniae* |  |
| *Yersinia aldovae* | *Streptococcus porcinus* |  |
| *Yersinia enterocolitica* | *Streptococcus pseudoporcinus* |  |
| *Yersinia frederiksenii* | *Streptococcus pyogenes* |  |
| *Yersinia intermedia* | *Streptococcus salivarius* |  |
| *Yersinia kristensenii* | *Streptococcus sanguinis* |  |
| *Yersinia pestis* | *Streptococcus sobrinus* |  |
| *Yersinia pseudotuberculosis* | *Streptococcus suis* |  |
| *Yersinia ruckeri* | *Streptococcus thoraltensis* |  |
| *Yokenella regensburgei* | *Streptococcus uberis* |  |
| *Streptococcus massiliensis* | *Streptococcus vestibularis* |  |
| *Streptococcus mitis* | *Treponema denticola* |  |
| *Streptococcus mutans* | *Treponema pallidum* |  |
| *Streptococcus oralis* | *Vagococcus fluvialis* |  |
| *Streptococcus ovis* | *Vibrio alginolyticus* |  |
| *Streptococcus parasanguinis* | *Vibrio cholerae* |  |
| *Streptococcus pasteurianus* | *Vibrio fluvialis* |  |
| *Streptococcus pluranimalium* | *Vibrio metschnikovii* |  |
| *Streptococcus pneumoniae* | *Vibrio mimicus* |  |
